# A structural explanation for the low effectiveness of the seasonal influenza H3N2 vaccine

**DOI:** 10.1371/journal.ppat.1006682

**Published:** 2017-10-23

**Authors:** Nicholas C. Wu, Seth J. Zost, Andrew J. Thompson, David Oyen, Corwin M. Nycholat, Ryan McBride, James C. Paulson, Scott E. Hensley, Ian A. Wilson

**Affiliations:** 1 Department of Integrative Structural and Computational Biology, The Scripps Research Institute, La Jolla, CA, United States of America; 2 Department of Microbiology, Perelman School of Medicine, The University of Pennsylvania, Philadelphia, PA, United States of America; 3 Department of Molecular Medicine, The Scripps Research Institute, La Jolla, CA, United States of America; 4 Department of Immunology and Microbiology, The Scripps Research Institute, La Jolla, CA, United States of America; 5 The Skaggs Institute for Chemical Biology, The Scripps Research Institute, La Jolla, CA, United States of America; Icahn School of Medicine at Mount Sinai, UNITED STATES

## Abstract

The effectiveness of the annual influenza vaccine has declined in recent years, especially for the H3N2 component, and is a concern for global public health. A major cause for this lack in effectiveness has been attributed to the egg-based vaccine production process. Substitutions on the hemagglutinin glycoprotein (HA) often arise during virus passaging that change its antigenicity and hence vaccine effectiveness. Here, we characterize the effect of a prevalent substitution, L194P, in egg-passaged H3N2 viruses. X-ray structural analysis reveals that this substitution surprisingly increases the mobility of the 190-helix and neighboring regions in antigenic site B, which forms one side of the receptor binding site (RBS) and is immunodominant in recent human H3N2 viruses. Importantly, the L194P substitution decreases binding and neutralization by an RBS-targeted broadly neutralizing antibody by three orders of magnitude and significantly changes the HA antigenicity as measured by binding of human serum antibodies. The receptor binding mode and specificity are also altered to adapt to avian receptors during egg passaging. Overall, these findings help explain the low effectiveness of the seasonal vaccine against H3N2 viruses, and suggest that alternative approaches should be accelerated for producing influenza vaccines as well as isolating clinical isolates.

## Author summary

Seasonal influenza vaccine does not always confer protection in vaccinated individuals. Vaccine candidates are selected from clinical isolates based on their antigenic properties. It is common to use chicken eggs for culturing clinical isolates and for large-scale production of vaccines. However, influenza virus often mutates to adapt to being grown in chicken eggs, which can influence antigenicity and hence vaccine effectiveness. Here, we structurally characterize an egg-adaptive substitution, namely L194P, in H3N2 virus hemagglutinin. Our results reveal that the L194P substitution substantially increases the flexibility of an epitope region that is commonly targeted by antibodies. Based on the binding affinity of a broadly neutralizing antibody and a panel of human serum antibodies, we further show that the L194P substitution dramatically changes the HA antigenicity. The change of the receptor-binding mode associated with the L194P substitution provides an explanation for its ability to successfully grow in eggs. Our study describes a mechanism for the low influenza vaccine effectiveness and reaffirms the urgency for replacing the egg-based production of influenza vaccines.

## Introduction

Recognition and neutralization of influenza virus by the immune system has been a subject of extensive research due to its profound implications for vaccine design. The majority of human antibodies against influenza virus that are elicited by natural infection or vaccination target the globular head domain of the hemagglutinin (HA) glycoprotein. In H3N2 viruses, five major antigenic sites A-E are the primary targets [1–3]. However, most of the globular head domain has an intrinsically high mutational tolerance [4, 5] that facilitates escape from the immune system. The receptor-binding site (RBS) is conserved but can still accommodate some level of mutation to evade antibody recognition. Rapid antigenic drift of influenza viruses can occur without perturbing vital functions and necessitates almost annual reformulation of the influenza vaccine.

Despite the first commercial influenza vaccines being approved in the US more than 70 years ago [6], complete and broad protection from an influenza vaccine has remained out of reach [7, 8]. Furthermore, in the past decade, the effectiveness of the seasonal vaccine against H3N2 viruses has been particularly low [8]. While the vaccine effectiveness was estimated to be 67% for seasonal H1N1 (pre-2009), 73% for H1N1pdm09, and 54% for type B, it was only 33% for H3N2 viruses [8]. Studies have attributed this low effectiveness of the H3N2 vaccine to the egg-based production process [9, 10]. Although eggs provide a cost effective way to grow influenza virus, the abundance of avian-type receptors on the chorioallantoic membrane [11, 12] often results in selection of variants that increase binding to avian-type receptors (NeuAcα2-3Gal), and reduce binding to human-type receptors (NeuAcα2-6Gal) [11, 13–16]. More importantly, these egg-adaptive substitutions on the HA have also been shown to impact antigenicity [17–22], leading to a decrease in vaccine effectiveness [9, 22]. The underlying mechanism is further elucidated by a recent structural study on RBS-targeted antibodies induced by an egg-adapted H1N1 vaccine strain [22]. Nonetheless, the structural and biophysical mechanisms involved in the antigenicity changes and the practical consequences to vaccine effectiveness resulting from egg-adaptive substitutions in H3N2 viruses remain to be fully explored.

Here, we performed a structural study on HA L194P, a commonly found egg-adaptive substitution in the H3N2 subtype. Previous studies have demonstrated that L194P changes the antigenicity [20], and also decreases the immunogenicity of H3N2 HA [19]. Here, *B*-value analysis of crystal structures with the L194P substitution revealed increased conformational dynamics of the 190-helix, and to a lesser extent the 150-loop, that explains the antigenic effect of L194P substitution on antigenic site B and its decreased affinity against an RBS-targeted broadly neutralizing antibody and human serum antibodies. Structural characterization further showed that the L194P substitution alters the receptor-binding mode and specificity. These structural and biophysical characterizations of the egg-adaptive substitution L194P therefore have important implications for the effectiveness of the seasonal vaccine against H3N2 viruses.

## Results

### Prevalence of HA L194P substitution in egg-passaged human H3N2 isolates

**Fig 1:**
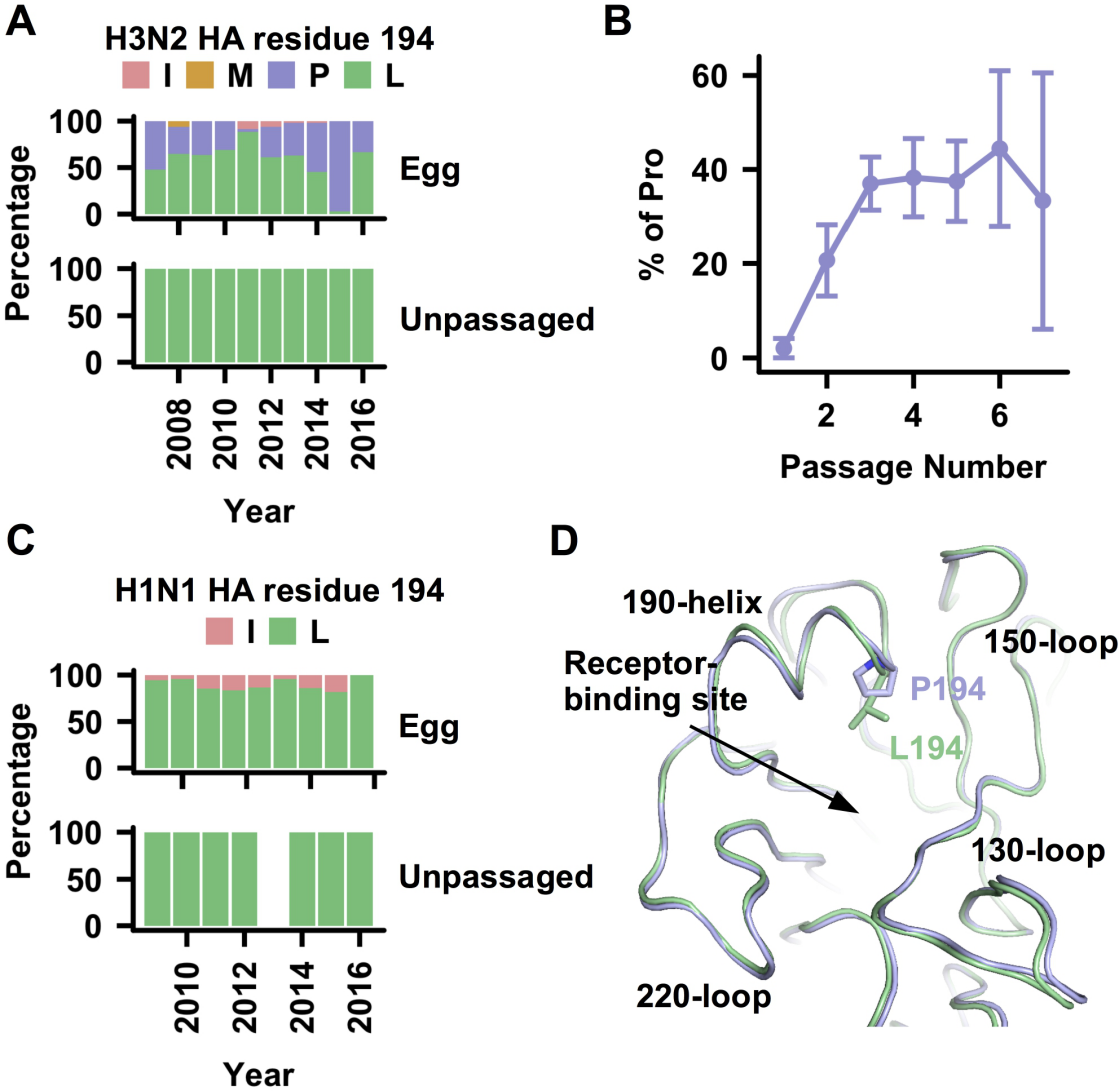
L194P is an egg-adaptive substitution in human H3N2 HA. **(A)** Frequency of different amino acids observed at HA residue 194 of human H3N2 isolates. Egg: isolates that were sequenced after passaging in egg. Unpassaged: isolates that were sequenced without passaging. **(B)** Egg-passaged human H3N2 isolates are categorized based on the number of passage. The fractions of isolates in different number of passage that possess a Pro at residue 194 are shown. Error bars represent the standard error estimated from a binomial distribution. **(C)** Frequency of different amino acids observed at HA residue 194 of pandemic H1N1 (“swine influenza”) human isolates. Egg: isolates that were sequenced after passaging in egg. Unpassaged: isolates that were sequenced without passaging. Of note, no sequence information was found for unpassaged isolates in 2013. **(D)** Cα traces of the HA structures of Bris07 P194 (blue) and L194 (green) are aligned and compared.

The HA L194P substitution rapidly emerges in human H3N2 viruses during passaging in chicken eggs [19, 23–25]. To examine the frequency of the L194P substitution in natural versus egg-passaged human viruses, the HA amino-acid sequences of human H3N2 isolates were retrieved from Global Initiative for Sharing Avian Influenza Data (GISAID; http://gisaid.org). We compared the HA sequences from human H3N2 isolates that were passaged in eggs versus isolates that were sequenced without any passaging (Fig 1A). Around 36% of egg-passaged human isolates possessed a Pro at residue 194, whereas all unpassaged human isolates contained Leu at residue 194. The frequency of the L194P substitution increased from ~2% after one passage in eggs to ~20% after two passages (Fig 1B), whereas after three or more passages, it stabilized around at ~37%. Interestingly, L194P is not observed in egg-passaged pandemic H1N1 (“swine flu”) human isolates (Fig 1C), suggesting that L194P is an H3N2-specific substitution. Subsequently, we focused on A/Brisbane/10/2007 (Bris07), a WHO-recommended H3N2 vaccine strain from 2008 to 2010 and a commonly employed model strain for experimental studies. There are 11 amino-acid sequences of Bris07 HA deposited in the NCBI protein database (S1 Table), one of which has an ambiguous amino acid at residue 194. For the other 10 sequences, five have Pro and the other five have Leu at residue 194. In fact, similar observations were made for the currently recommended H3N2 vaccine strains A/Hong Kong/4801/2014 (HK14) and A/Singapore/INFIMH-16-0019/2016 (Sing16). HK14 is a recommended H3N2 vaccine strain for the 2016–2017 and 2017–2018 northern hemisphere influenza seasons, and also for the 2016 and 2017 southern hemisphere influenza seasons, whereas Sing14 is a recommended H3N2 vaccine strain for the 2018 southern hemisphere influenza season. In the eight amino-acid sequences of HK14 HA deposited in GISAID (S2 Table), four have Pro and four have Leu at residue 194. Similarly, in the four amino-acid sequences of Sing16 HA deposited in GISAID (S2 Table), three have Pro and one has Leu at residue 194. Together, these observations clearly underscore the importance of structural and functional characterization of the L194P substitution in the context of its ramifications for vaccine development.

### HA L194P substitution increases conformational dynamics

Previous studies have shown that the HA L194P substitution can affect antibody binding to antigenic site B [19, 20], which is composed of the top of the 190-helix and part of the 150-loop. To understand the structural implications of this mutation, we determined crystal structures of Bris07 HA with both Leu (L194) and Pro (P194) at residue 194 to 1.70 Å and 2.30 Å, respectively (S3 Table). Except for a slight displacement (less than 1.0 Å) of the 190-helix and the 130-loop, the backbone conformations of Bris07 P194 and L194 are almost identical (Fig 1D). Thus, the effect of the L194P substitution on antigenicity is not simply due to alteration of the overall structural conformation or of the amino-acid side-chain substitution *per se*. Rather, we hypothesized that its impact on HA antigenicity might also be exerted through some effects on the mobility of the HA structure as reflected by its conformational dynamics.

**Fig 2:**
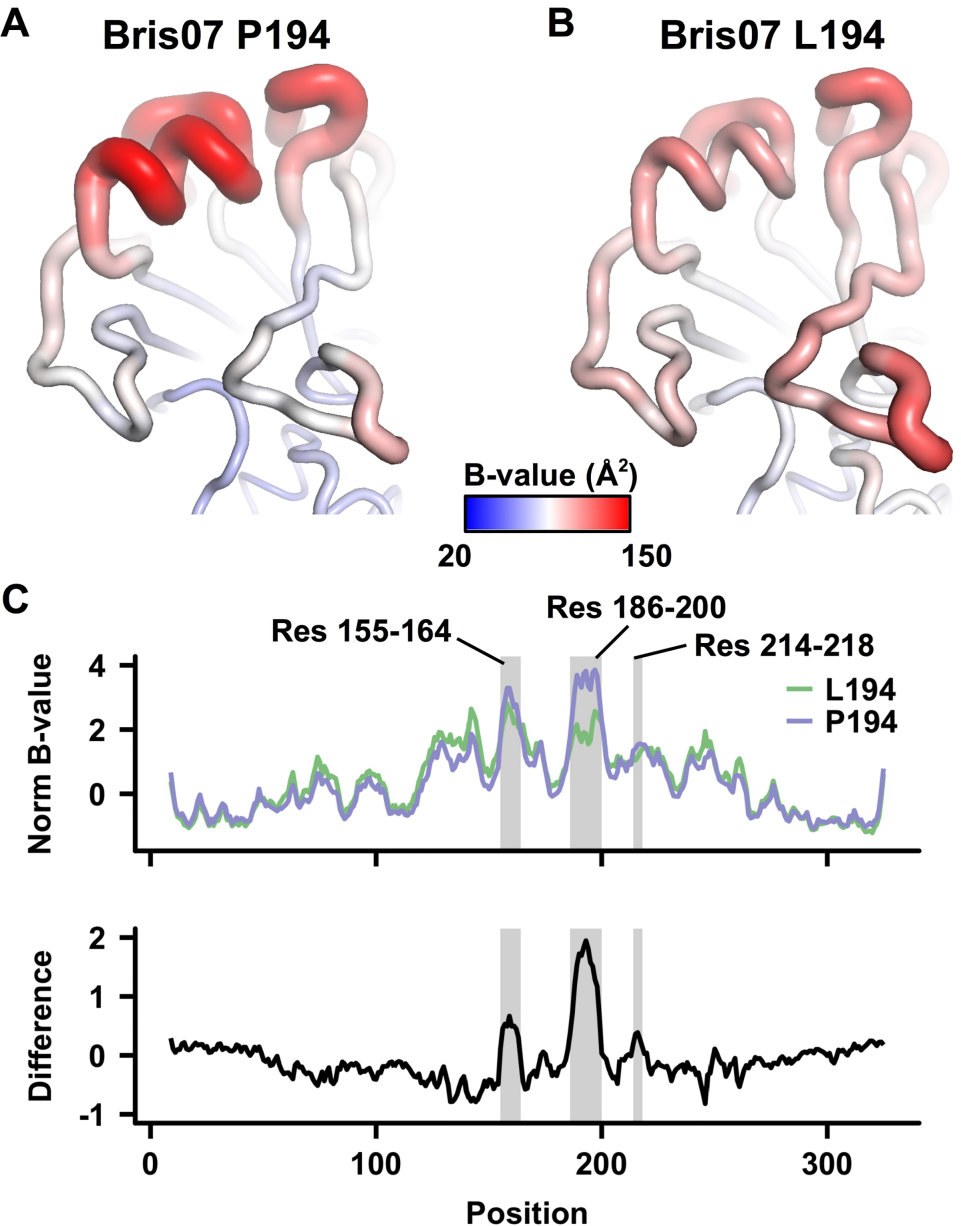
*B*-value analysis of Bris07 HA. **(A-B)** *B*-values (Å^2^) of Cα atoms in **(A)** Bris07 P194 and **(B)** Bris07 L194 are projected on the HA structure. Of note, on average, the *B*-values of Cα atoms in Bris07 P194 (mean ± s.d. = 58 ± 24 Å^2^) are lower than that of Bris07 L194 (mean ± s.d. = 64 ± 24 Å^2^). **(C)** The normalized *B*-values of Cα atoms in Bris07 P194 (blue) and L194 (green) are compared. The bottom panel shows their difference in HA1: (normalized *B*-values of Cα atoms in P194)–(normalized *B*-values of Cα atoms in L194). The amino-acid position is plotted along the x-axis. Positions corresponding to the residues (Res) of interest are shaded in grey.

One way to assess difference in mobility is to compare the thermal displacement values as reflected by the *B*-values of the Cα atoms in the wild-type and L194P HA crystal structures. In this way, we can investigate any effect of the L194P substitution on either local or global conformational dynamics of the HA. To minimize any possible experimental bias, we obtained another X-ray structure for Bris07 L194 that was crystallized in the same precipitant condition as Bris07 P194 and diffracted to a similar resolution (2.35 Å) to that of Bris07 P194 (S3 Table). While the overall average *B*-value of Cα atoms in Bris07 P194 (58 Å^2^) was slightly lower than that of Bris07 L194 (64 Å^2^), the 190-helix (residues 188 to 197) in Bris07 P194 (Fig 2A) was notably higher (142 Å^2^) than that of Bris07 L194 (109 Å^2^) (Fig 2B). This difference could be visualized by comparing the electron density in the 2Fo-Fc maps, which was much weaker for the 190 helix of Bris07 P194 compared to that of Bris07 L194 (S1 Fig). The mobility of the 190-helix in Bris07 P194 was also captured in a molecular dynamics simulation (S2 Fig). We further compared the normalized *B*-values of Cα atoms from the two structures (see Materials and Methods). By plotting the difference in normalized *B*-values against the amino-acid residue position, one major peak and two minor peaks were observed (Fig 2C). The major peak is centered around residue 193 (range = residues 186 to 200), which corresponds to the 190-helix region. The first minor peak is centered on residue 159 (range = residues 155 to 164), which corresponds to the 150-loop region. The second minor peak is centered on residue 217 (range = residues 214–218), corresponding to a region facing towards the trimer axis that is proximal to the 190-helix (S3 Fig). The major peak reaffirms that L194P increases the conformational dynamics of the 190-helix, whereas the minor peaks suggest that the increase in conformational dynamics propagates to some extent to the neighboring regions.

### Antigenicity change resulting from HA L194P substitution

**Fig 3:**
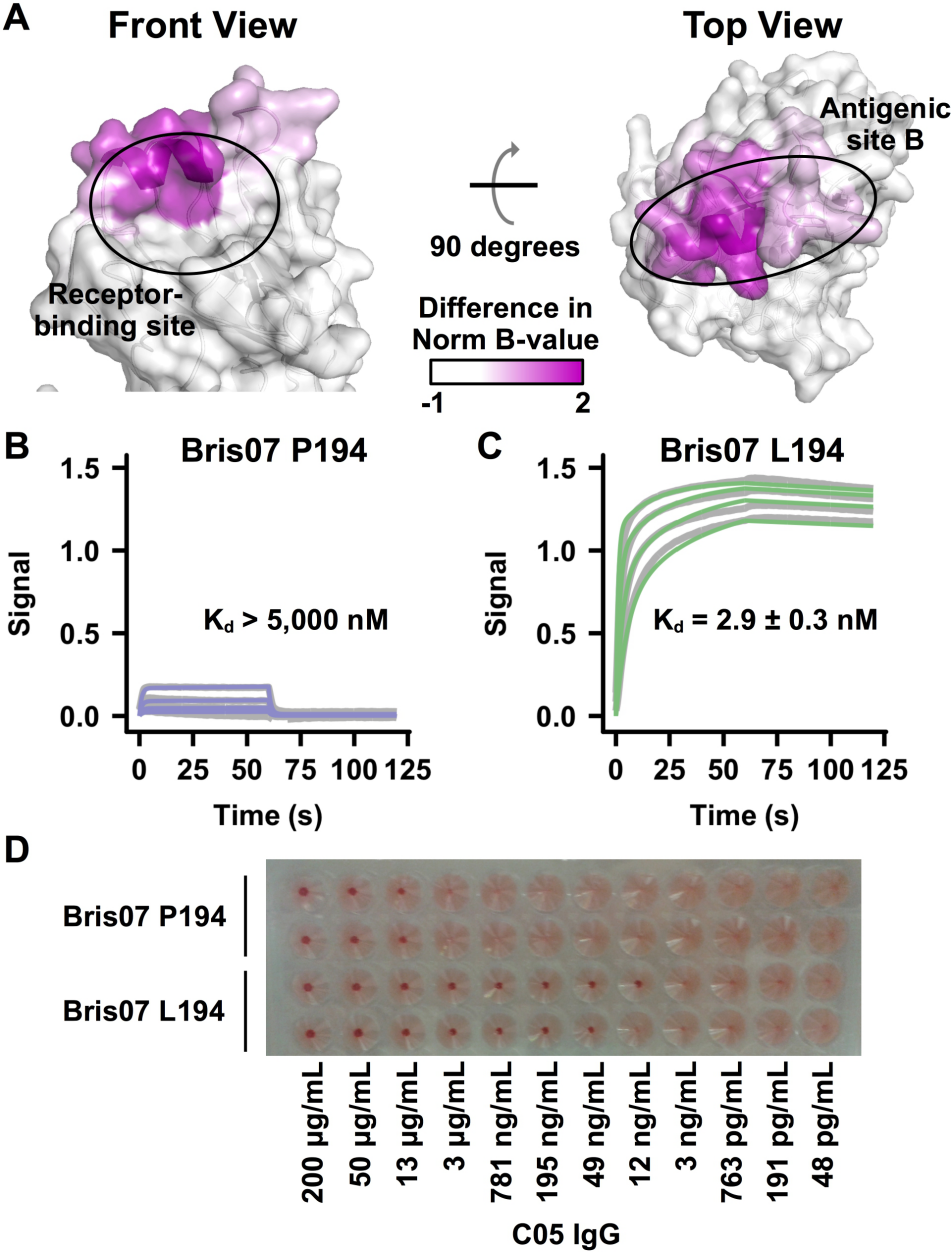
Impact of L194P substitution on antigenicity. **(A)** The difference in normalized *B*-values between Bris07 P194 and Bris07 L194 (B-value Diff.) for each Cα atom is projected on the HA structure of Bris07 L194. *B*-value Diff. = (normalized *B*-values of Cα atoms in P194)–(normalized *B*-values of Cα atoms in L194). **(B-C)** Biolayer interferometry (BLI) was used to measure the binding kinetics of C05 IgG against the recombinant HA proteins of **(B)** Bris07 P194 and **(C)** Bris07 L194. **(D)** HAI assay of C05 IgG against Bris07 P194 and Bris07 L194 viruses. Duplicates were performed for each of Bris07 P194 and L194.

The region with higher conformational dynamics in Bris07 P194 includes part of the RBS and antigenic site B (Fig 3A). While previous studies have shown that the L194P substitution can affect antibody binding to antigenic site B [19, 20], we also demonstrate here that it also affects the binding of RBS-targeted broadly neutralizing antibody C05 [26]. The K_d_ of C05 IgG for Bris07 L194 was around 3 nM, but for Bris07 P194 was much weaker at >5 μM, as measured by a biolayer interferometry binding assay (Fig 3B and 3C). Moreover, C05 IgG exhibited a minimum inhibitory concentration of 12–49 ng mL^-1^ against Bris07 L194 virus, but only 13 μg mL^-1^ against Bris07 P194 virus as measured in a hemagglutination inhibition assay (Fig 3D). Since residue 194 is also part of the C05 epitope [26], the three orders of magnitude diminishment in binding and neutralization is likely due to a combination of elevated conformational dynamics in the RBS and the altered epitope structure.

**Fig 4:**
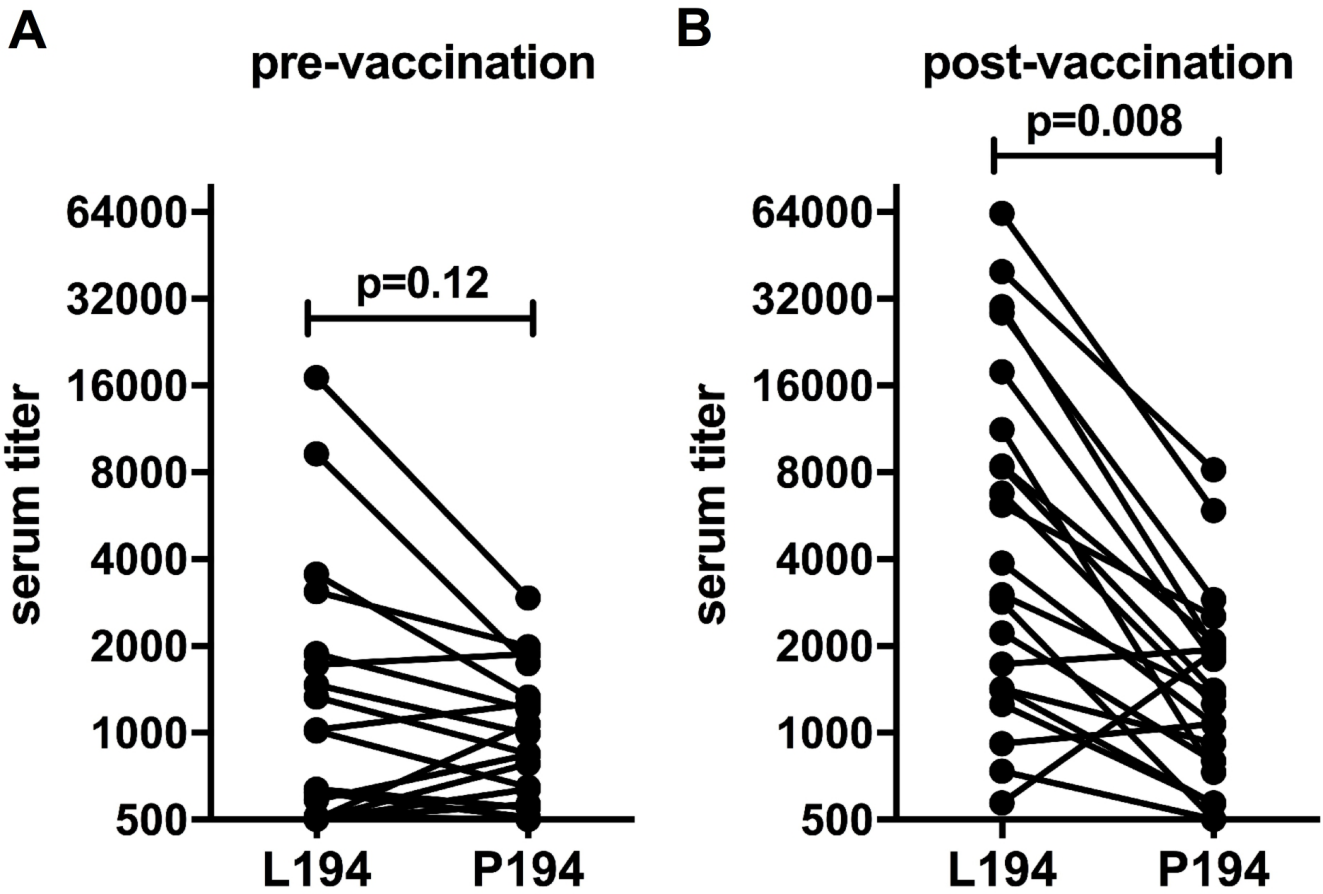
Vaccines elicit antibodies in humans that poorly react to HA with the L194P mutation. Sera from 21 human donors were collected before **(A)** and after **(B)** vaccination with the 2015–2016 influenza vaccine. Antibody binding to virus-like particles expressing WT and L194P HA was measured using ELISA. Titer is expressed as the dilution of sera required to reach a fixed absorbance (O.D. = 0.75). The geometric mean ELISA titers calculated after testing each sera sample in triplicate are shown. Significance was determined using a paired Student’s t-test.

Furthermore, we also observed that the antigenicity of A/Switzerland/9715293/2013 (Switz13) HA, which was the H3 component of the 2015–2016 seasonal influenza vaccine, was influenced by L194P substitution. We obtained human serum samples from 21 donors before and after vaccination with the 2015–2016 seasonal influenza vaccine, which carries an Leu at residue 194 in the seed stock. The binding of antibodies in these serum samples to HAs of both Switz13 WT and L194P was tested using ELISAs (Fig 4, S4 Fig and S5 Fig). Before vaccination, the binding of serum antibodies to Switz13 WT (mean serum titer = 2,253) was higher than to Switz13 L194P (mean serum titer = 1,037), although such difference was not statistically significant (p = 0.12, paired Student’s t-test). The lack of significant difference is likely attributed to the binding of antibodies to epitopes other than site B. After vaccination, the binding of serum antibodies to Switz13 WT (mean serum titer = 11,452) was boosted and was significantly higher than to Switz13 L194P (mean serum titer = 1,903; p = 0.008, paired Student’s t-test). This difference is likely due to a boost in antibodies that target antigenic site B (with L194), which is immunodominant in recent human H3N2 viruses [20, 27], and which does not cross-react with the Switz13 L194P. Overall, these results confirm that L194P substitutions alter the antigenicity of H3 HA.

### Receptor binding mode and specificity of HA L194P substitution

**Fig 5:**
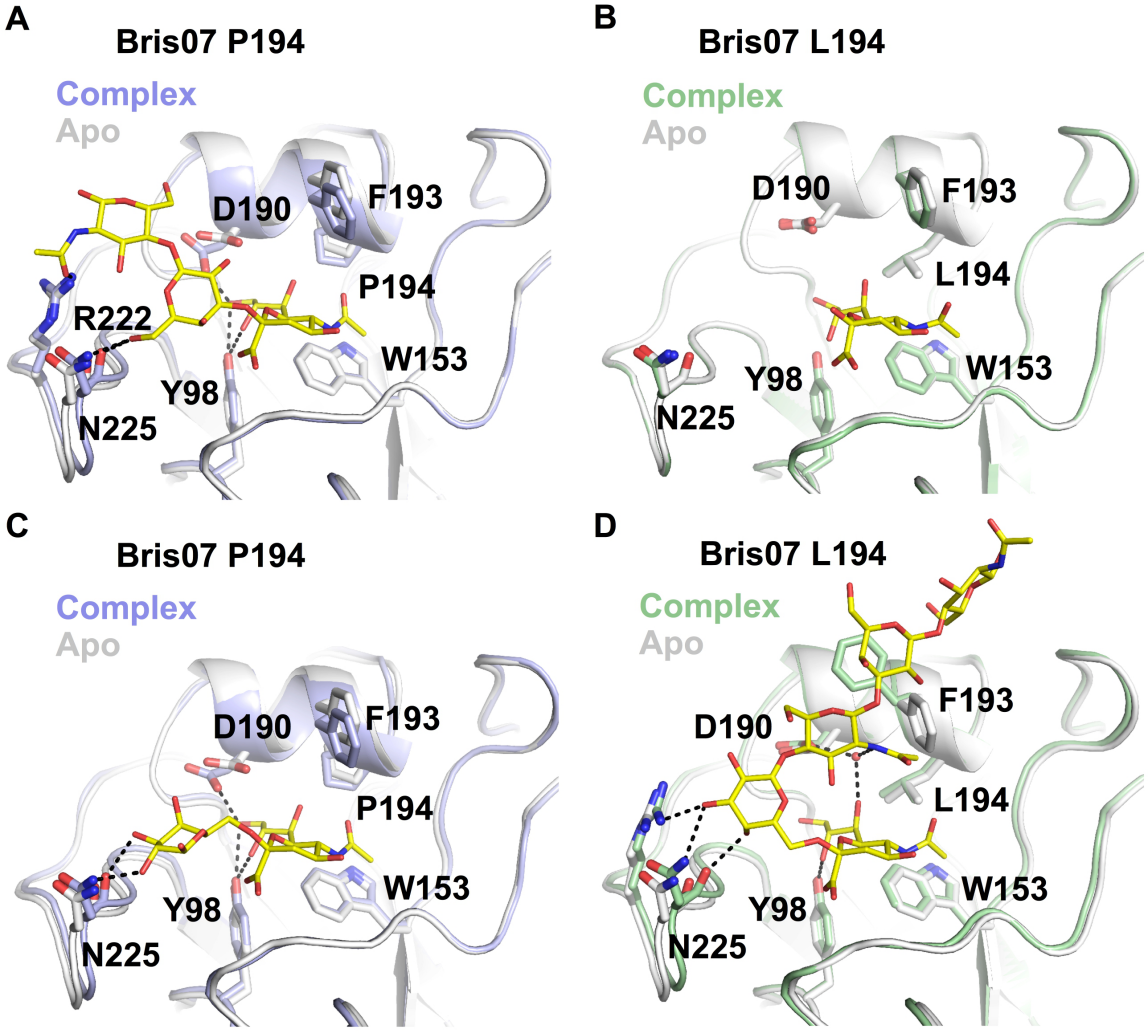
Crystal structures of Bris07 P194 and L194 HAs in complex with receptor analogs. HA structures of **(A)** Bris07 P194 in complex with 3'SLNLN, **(B)** Bris07 L194 in complex with 3'SLNLN, **(C)** Bris07 P194 in complex with 6'SLNLN, and **(D)** Bris07 L194 in complex with 6'SLNLN are shown. The apo form for each structure is aligned and colored in grey. Glycan receptor analogs (3'SLNLN and 6'SLNLN) are colored in yellow and shown as stick representations. Hydrogen bonds are represented by black dashed lines.

We further examined the impact of L194P substitution on receptor binding. Glycan array analysis showed that Bris07 L194 HA has typical strong human-type receptor specificity. In contrast, binding avidity of the P194 variant was generally weak, with negligible binding to the glycan array (S6 Fig). Crystal structures of Bris07 L194 and P194 in complex with avian receptor analog 3’SLNLN (NeuAcα2-3Galβ1-4GlcNAcβ1-3Galβ1-4GlcNAc) and human receptor analog 6’SLNLN (NeuAcα2-6Galβ1-4GlcNAcβ1-3Galβ1-4GlcNAc) were determined at 1.75 to 2.3 Å resolutions with 100-fold molar excess of ligand (S4 Table). The 3’SLNLN adopts a typical *trans* conformation [28] in complex with Bris07 P194 (Fig 5A), but the 190-helix is slightly lower and the 220-loop shifted such that the RBS is more compact compared to the apo form. For Bris07 L194, the binding mode of 3’SLNLN could not be confidently determined due to poor electron density for the 3’SLNLN (Fig 5B and S7 Fig). The electron density for 3’SLNLN in Bris07 P194 allowed us to model three monosaccharide moieties (Sia-1, Gal-2, and GlcNAc-3), whereas that in Bris07 L194 only permitted us to barely model Sia-1. Taken together, these results suggest that the binding affinity against the avian receptor is increased, albeit not dramatically, in the presence of L194P substitution, which is consistent with its role in egg adaption.

When binding to human receptor analog 6’SLNLN, Bris07 P194 exhibits a similar conformational change in the 190-helix and 220-loop as seen in its 3’SLNLN-bound form (Fig 5A and 5C). Only the first two monosaccharide moieties (Sia-1 and Gal-2) of 6’SLNLN that bind to Bris07 P194 were ordered as evaluated from the electron density map (S7 Fig). Interestingly, their conformation is similar to the human receptor analog in complex with the avian H5 HA [29], where Gal-2 is rotated by 90° as compared to the energetically favorable folded-back conformation [30], while maintaining a *cis* configuration (S8 Fig).

In contrast, 6’SLNLN binds to Bris07 L194 in the folded-back conformation (Fig 5D). Binding to 6’SLNLN causes a slight shift of the 220-loop in Bris07 L194, similar to that in Bris07 P194. However, unlike Bris07 P194, the backbone of 190-helix in Bris07 L194 maintains at the same position when binding to 6’SLNLN such that D190 forms water-mediated hydrogen bonds with Sia-1 and GlcNAc-3 (Fig 5D). In addition, when binding to 6’SLNLN, F193 side chain of Bris07 L194 but not of Bris07 P194 adopts a different rotamer. The F193 side chain points towards the RBS in the Bris07 L194 apo form, whereas it points away from the RBS in the 6’SLNLN-bound form. The change in the F193 rotamer is therefore critical for 6’SLNLN binding, as the rotamer observed in the apo form would sterically clash with GlcNAc-3. In addition, the aromatic ring of F193 stabilizes Gal-4 by a carbohydrate-aromatic CH-π stacking interaction [31] when it points away from the RBS. This CH-π stacking interaction is not present in ancestral A/Hong Kong/1/1968 (HK68) HA [32], which has a Ser at residue 193. Comparison to the structure of HK68 HA in complex with human receptor analog reveals that such a stacking interaction shifts the location of Gal-4 towards the top of the RBS (S9 Fig). Subsequently, this displacement of Gal-4 may prevent a steric clash between GlcNAc-5 and F159. Nevertheless, the driving force for such evolutionary changes and its functional consequences are outside of the scope of this study and require further investigation. Overall, these structure analyses show that L194P substitution disfavors binding to human receptors as a result of a significant change in the receptor-binding mode.

The elevated mobility as assessed by conformational dynamics in the 190-helix of Bris07 P194 apo form appears to be diminished in the receptor-bound forms, as suggested by normalized *B*-value analysis (S10 Fig). This implies that L194P substitution increases the entropic cost of receptor binding, which in turn may explain the generally low avidity against sialylated glycans (S6 Fig). At the same time, the L194P substitution results in lowering of 190-helix such that a hydrogen bond can be formed between D190 and Sia-1 (Fig 5A and 5C). This additional favorable interaction may help alleviate the entropic cost during receptor binding.

## Discussion

It should be noted that the receptor specificity changes conferred by the L194P substitution differ from the traditional ones that facilitate avian-to-human transmission where substantial diminishment in binding to avian receptors is accompanied by a large affinity gain towards human receptors [33, 34]. The atypical receptor specificity change observed in L194P is likely due to the glycan content in embryonated chicken eggs. Firstly, sialylated glycans in eggs are short and are predominantly α2–3 linkage [12], whereas recent human H3N2 viruses have a receptor specificity favoring long α2–6 sialylated glycans with several LacNAc repeats [35]. Therefore, passaging human H3N2 viruses in eggs would result in a strong selection against α2–6 sialylated glycans. Secondly, there may be a high surface density of sialylated glycans in eggs, as suggested by the fact that eggs can support a wild-type-level growth of an RBS mutant Y98F that has a low receptor-binding affinity and is attenuated in vivo [36, 37]. These observations, along with the results from our study, indicate that a slight increase in binding towards avian receptors is sufficient to improve replication efficiency of human H3N2 viruses in eggs.

Change in antigenicity caused by egg-adaptive substitutions is common for influenza virus [17–20, 22]. Such an antigenic effect was expected to only impact the epitopes that contain the substitution of interest [22]. However, we demonstrate here that the egg-adaptive substitution L194P in H3 subtype disrupts a larger region spanning the 190-helix and part of the 150-loop in the HA RBS. In fact, such conformational dynamics has been shown to influence antigenicity in several other pathogens [38–40]. While this type of effect caused by the L194P substitution may not be as applicable to other egg-adaptive substitutions, it may have profound implications for vaccine effectiveness. The prevalence of the L194P substitution from egg-passaged H3N2 viruses will likely hinder the ability of influenza vaccine to induce an antibody response that effectively protects against circulating seasonal H3N2 viruses, especially for responses against antigenic site B and surrounding epitope regions. Importantly, antigenic site B has been shown to be immunodominant in recent human H3N2 viruses [20, 27] and hence such a substitution would have a more profound effect on the antibody response. The extensive usage of chicken eggs for passaging clinical isolates and for vaccine production suggests that the egg-adaptive substitution L194P may contribute to the low vaccine effectiveness against H3N2 subtype [8]. A recent study indeed demonstrated that an egg-adaptive substitution Q226R in H1N1 vaccine elicits an immune response that preferentially targets the vaccine strain, but not its circulating counterpart [22]. As annual vaccination remains the major preventive measure against influenza virus, it may be beneficial to accelerate consideration of alternative approaches for influenza vaccine production [41] to optimize the protective effectiveness of the vaccine [22].

## Materials and methods

### Sequence analysis

A total of 28,694 full-length human H3N2 HA protein sequences and 21,081 full-length pandemic H1N1 (“swine influenza”) HA protein sequences were downloaded from the Global Initiative for Sharing Avian Influenza Data (GISAID; http://gisaid.org). Sequences with ambiguous amino acids were removed. Sequence alignment was performed by MAFFT version 7.157b [42]. Passaging history was determined by parsing regular expression in FASTA headers as described [43]. For human H3N2 HA, a total of 349 egg-passaged sequences and 853 unpassaged sequences from year 2007 and later were included in our analysis. For pandemic H1N1 HA, a total of 585 egg-passaged sequences and 631 unpassaged sequences from year 2009 and later were included in our analysis. The 11 Bris07 sequences from the NCBI protein database (https://www.ncbi.nlm.nih.gov/protein/) were obtained by searching “A/Brisbane/10/2007, hemagglutinin”. The eight HK14 and four Sing16 sequences from GISAID were obtained by searching “A/Hong Kong/4801/2014” and “A/Singapore/INFIMH-16-0019/2016”, respectively, in the query result of all human H3N2. Only those strains with a strain name that was identical to “A/Hong Kong/4801/2014” for HK14 or to “A/Singapore/INFIMH-16-0019/2016” for Sing16 were analyzed. All computer scripts for this analysis have been deposited to https://github.com/wchnicholas/H3N2L194P.

### Expression, crystallization and structural determination

HA and C05 IgG expression and purification were performed as described [26]. Initial HA crystal screening was carried out using our high-throughput, robotic CrystalMation system (Rigaku, Carlsbad, CA) using the sitting drop vapor diffusion method at 20°C. Further refinement of the conditions obtained from the initial hits was performed by sitting drop vapor diffusion at 20°C with a 500 μL reservoir solution and each drop consisting of 0.8 μL protein + 0.8 μL precipitant. To generate HA-receptor complexes, crystals were soaked in reservoir solution supplemented with 20 mM of receptor analogs for 2 hours. The resulting crystals were flash cooled, and stored in liquid nitrogen until data collection. The crystallization conditions are described in S5 Table. Diffraction data were collected at the APS GM/CA-CAT 23ID-D and at the Stanford Synchrotron Radiation Lightsource beamline 12–2. The data were indexed, integrated and scaled using HKL2000 (HKL Research, Charlottesville, VA) [44]. The structure was solved by molecular replacement using Phaser [45] with PDB: 4O5N [46] as the molecular replacement model, modeled using Coot [47], and refined using Refmac5 [48]. Ramachandran statistics were calculated using MolProbity [49].

### Molecular dynamics simulation

Residues 57–263 from monomeric HA1 were used as the starting structure. The starting structure was parameterized using Amber ff14SB force field [50] and GLYCAM06 force field [51]. Chlorine counterions were added to neutralize the charge and the system was solvated with the TIP4P-Ew water model [52] in a cubic box with 10 Å between the solute surface and the box boundary. Periodic box boundary conditions were implemented. Amber16 [53] was used for energy-minimization, heating and equilibration. Energy-minimization was performed sequentially by 1000 steps of hydrogen-only minimization, 4000 steps of solvent minimization, 2000 steps with the protein backbone constrained, and 5000 steps of all atom minimization. Heating was performed by molecular dynamics simulation with a 2 fs time-step from 0 K to 300 K linearly over 250 ps in the NVT ensemble. Position restraints of 5.0 kcal mol^-1^ Å^-2^ were used on the protein backbone atoms. Temperature was controlled using the Langevin thermostat with a collision frequency of 1 ps^-1^. Equilibration was performed at 300 K in the isothermal-isobaric (NPT) ensemble, first with position restraints for 500 ps and a relaxation time of 2 ps, and then without position restraints for 500 ps.

Production runs of 520 ns were performed in the NPT ensemble at 300 K using the pmemd.cuda module of Amber16 [53] and the Langevin thermostat with a collision frequency of 5 ps^-1^. Constant pressure was monitored using the Berendsen barostat with a pressure relaxation time of 2 ps. Hydrogen mass repartitioning [54] was used in all the simulations, allowing time-steps of 4 fs instead of 2 fs. Root-mean-square fluctuations (RMSF) of Cα atoms and simulated *B*-values were calculated in CPPTRAJ [55] for all residues from aligned trajectories after discarding the first 20 ns of the production run.

### Normalized *B*-value analysis

The *B*-values of the Cα atoms were normalized as previously described [56] using the following equation:

$$B^{\prime }=\frac{B-\langle B\rangle }{\sigma (B)}$$

where *B*′ is the normalized *B*-value, *B* is the measured *B*-value, 〈*B*〉 is the average value of all measured *B*-values for Cα atoms, and σ(*B*) is the standard deviation of all measured *B*-values for Cα atoms. Of note, while the *B*-value has a unit of Å^2^, the normalized *B*-value is unit free.

### Biolayer interferometry binding assay

An Octet Red instrument (ForteBio, Menlo Park, CA) was employed for the biolayer interferometry binding assay. C05 IgG at a concentration of 50 μg mL^-1^ was loaded onto the anti-human IgG Fc Capture (AHC) Biosensors. Binding kinetics were measured against the indicated HA at 250 nM, 500 nM, 1,000 nM, and 2,000 nM. The data were fit with a 1:2 bivalent model to estimate the K_d_.

### Hemagglutination inhibition assay

Influenza viruses carrying the Bris07 HA were rescued by the eight-plasmid reverse genetic system [57], using six internal genes from A/WSN/33, NA from A/Hong Kong/1/1968, and HA from Bris07. Turkey red blood cells (Lampire Biological Laboratories, Pipersville, PA) were used for measuring the HA titer. Hemagglutination inhibition assay was then set up using four hemagglutinating units of virus as previously described [26].

### Virus-like particles for ELISAs

Virus-like particles (VLPs) were harvested after transfecting 293T cells with plasmids encoding human-airway trypsin-like protease (HAT), HIV gag, an irrelevant neuraminidase, and influenza HA. Three days post-transfection, supernatants were collected and centrifuged to remove cell debris. Supernatants were then concentrated by ultracentrifugation in a SW-28 rotor at 19,000 RPM with a 20% sucrose (w/vol) cushion and resuspended in phosphate-buffered saline (PBS).

### Serum antibody ELISAs

Human serum samples were obtained from 21 donors prior to vaccination and at least 28 days following vaccination with the 2015–2016 seasonal influenza vaccine. ELISA plates were coated overnight at 4°C with VLPs expressing Switz13 WT HA (the H3 component of the 2015–2016 vaccine) or Switz13 with an L194P HA mutation. The following day, plates were blocked with a 3% (w/vol) bovine serum albumin (BSA) in PBS for 2 hours. Plates were washed 3 times with PBS containing 0.1% Tween20 (vol/vol) and serial dilutions of each serum sample in ELISA buffer (1% BSA w/vol in PBS) were added to plates. Plates were again washed after 2 hours of incubation at room temperature and an anti-human secondary Ab conjugated to a peroxidase (Jackson ImmunoResearch) was added in ELISA buffer. Plates were washed after incubating with the secondary Ab for 1 hour at room temperature, after which a 3,3’,5,5’-tetramethylbenzidine (TMB) substrate (Seracare) was added to each plate. Absorbance was measured after stopping the reaction with HCl. After subtracting background values, one-site specific binding curves were fitted for each serum sample using the Prism software package. Using the curve fit, the dilution of serum required to give a fixed absorbance (OD = 0.75) was calculated for each sample against both WT and L194P VLPs. ELISAs were repeated in three independent experiments, each on separate days, and the geometric mean of the replicate titers is reported for each individual.

### Glycan array analysis

Recombinant trimeric HA0 was expressed in HEK293S GnTI^-/-^ (American Type Culture Collection), purified and analyzed on glycan array as previously described [35]. Briefly, soluble trimeric HA (50 μg mL^-1^) was pre-complexed with the anti-HIS mouse antibody (MA1-21315, Thermo Fisher Scientific, Waltham, MA) and the Alexa647-linked anti-mouse IgG (A-21235, Thermo Fisher Scientific) at 4:2:1 molar ratio for 15 min on ice in 50 μL PBST. This complex was incubated on the array surface in a humidified chamber for 60 min before washing and analysis using an Innoscan 1100AL microarray scanner (Innopsys, Chicago, IL). Fluorescent signal intensity was measured using Mapix (Innopsys) and mean intensity minus mean background of 4 replicate spots was calculated. A complete list of the glycans on the array is presented in S11 Fig.

### Ethics statement

Human serum samples were obtained with informed consent and with the approval of the Wistar Institute Institutional Review Board. Experiments with de-identified human serum samples were conducted at the University of Pennsylvania with the approval of the University of Pennsylvania Institutional Review Board.

## Supporting information

S1 Table Variants at residue 194 of Bris07 HA sequences from the NCBI protein database. (PDF)

S2 Table Variants at residue 194 of HK14 and Sing16 HA sequences from the GISAID. (PDF)

S3 Table X-ray data collection and refinement statistics for Bris07 Apo HAs. (PDF)

S4 Table X-ray data collection and refinement statistics for Bris07 HA in complex with receptor analogs. (PDF)

S5 Table Crystallization conditions. (PDF)

S1 Fig Final 2Fo-Fc electron density maps of the receptor binding sites of Bris07 P194 and L194 HAs. Final 2Fo-Fc electron density maps for the backbone of the receptor binding sites (grey sticks) are represented in a green mesh and contoured at the indicated σ levels to illustrate differences in electron density levels between Bris07 P194 and L194 HAs.
(PDF)

S2 Fig Molecular dynamics simulation of Bris07 P194 and L194 HAs. **(A)** 50 random frames from the 500 ns molecular dynamics simulation for Bris07 P194 (blue) and L194 (green) are aligned. **(B)** The simulated *B*-values of Cα atoms in Bris07 P194 and L194 are compared. The bottom panel shows their difference: (simulated *B*-values of Cα atoms in P194)–(simulated *B*-values of Cα atoms in L194). Positions corresponding to the residues (Res) of interest are shaded in grey. The positions of three sharp inverse peaks are indicated. Of note, these inverse peaks were not observed in normalized *B*-value analysis (Fig 2C). **(C)** Those residues (orange) corresponding to the inverse peaks in panel B, along with P194 (blue), are shown on the HA trimer structure. One protomer of the HA trimer is shown in cartoon representation and the other two protomers are shown in surface representation. Those three residues (D104, S199, and R208) are either involved in or close to the interface between protomers. Therefore, we rationalized that these inverse peaks in panel B represent an artifact from molecular dynamics simulation due to the usage of a monomeric instead of trimeric structure.
(PDF)

S3 Fig Elevated conformational dynamics of the 190-helix proximity region that faces towards the trimer axis. The locations of residues 214–218, which have a higher normalized *B*-value in Bris07 P194 as compared to Bris07 L194, are highlighted in red in all three protomers of the HA trimer. One protomer is shown in cartoon representation and the other two protomers are shown in surface representation. P194 is shown as stick representation and colored blue.
(PDF)

S4 Fig Complete binding curves for pre-vaccination human serum samples against VLPs with Switz13 WT and L194P HAs. Error bars for individual points represent the standard error of the mean (SEM), while dashed lines represent the 95% confidence interval of the one-site specific binding curve fit to the ELISA data (N = 3 experimental replicates).
(PDF)

S5 Fig Complete binding curves for post-vaccination human serum samples against VLPs with Switz13 WT and L194P HAs. Error bars for individual points represent the standard error of the mean (SEM), while dashed lines represent the 95% confidence interval of the one-site specific binding curve fit to the ELISA data (N = 3 experimental replicates).
(PDF)

S6 Fig Glycan array analysis of recombinant HA from Bris07 P194 and L194. 293S-expressed recombinant HA was purified and analyzed by the sialoside glycan array.
(PDF)

S7 Fig Final 2Fo-Fc and omit (Fo-Fc) electron density maps of glycan receptor analogs (3'SLNLN or 6'SLNLN) in complex with Bris07 P194 and L194 HAs. Final 2Fo-Fc electron density maps for the glycan receptor analogs (yellow sticks) are represented in a green mesh and contoured at 0.9 σ (left). Omit (Fo-Fc) electron density maps for the glycan receptor analogs are represented in a green mesh and contoured at 2.0 σ (right).
(PDF)

S8 Fig Conformations of human receptor analog in complex with avian H5 HAs and with Bris07 P194 HA. The structures of A/turkey/Turkey/1/2005 H5 HA in complex with 6'SLN (PDB 4BH0 [29]) and A/Vietnam/1194/2004 H5 HA in complex with 6'SLN (PDB 4BGX [29]) are aligned to the structure of Bris07 P194 in complex with 6'SLNLN. HAs are shown in cartoon representations. The first two monosaccharide moieties of the human receptor analogs (6'SLN and 6'SLNLN) are shown in stick representations.
(PDF)

S9 Fig Comparison between the conformations of human receptor analogs in Bris07 L194 HA and in HK68 HA. The structure of Bris07 L194 in complex with human receptor analog 6'SLNLN is aligned with the structure of HK68 HA in complex with human receptor analog sialylneolacto-N-tetraose c (PDB 2YPG [32]) based on the receptor-binding subdomain (residues 117–265 [58]).
(PDF)

S10 Fig *B*-value analysis of Bris07 P194 HA in receptor-bound forms. **(A)** The normalized *B*-values of Cα atoms in Bris07 P194 apo form, 3'SLNLN-bound form, 6'SLNLN-bound form are compared. **(B)** The difference between the normalized *B*-value in apo and 3'SLNLN-bound forms is shown. Difference = (normalized *B*-values of Cα atoms in apo form)–(normalized *B*-values of Cα atoms in 3'SLNLN). **(C)** The difference between the normalized *B*-value in apo and 6'SLNLN-bound forms is shown. Difference = (normalized *B*-values of Cα atoms in apo form)–(normalized *B*-values of Cα atoms in 6'SLNLN). The amino-acid position is plotted along the x-axis. Positions corresponding to the residues (Res) of interest are shaded in grey.
(PDF)

S11 Fig Glycan array compound list. The name and structure of each compound on the glycan array are shown.
(PDF)
