# Supplementary material for: A structural explanation for the low effectiveness of the seasonal influenza H3N2 vaccine
Source: PLoS Pathog. 2017 Oct 23;13(10):e1006682. doi: 10.1371/journal.ppat.1006682 (PMC5667890; doi:10.1371/journal.ppat.1006682)
Supplement: S1 Table — (PDF) [file ppat.1006682.s001.pdf]

Accession	Residue 194[a]
AFN11834.1	P
ABW23424.1	L
ABW23422.1	P
ADI44916.1	X
AIW60702.1	L
AIW60701.1	P
AHX37617.1	P
AIU46080.1	L
ABW23353.1	P
ACO95270.1	L
ACI26318.1	L

[a]X indicates ambiguous amino acid
