# Supplementary material for: A structural explanation for the low effectiveness of the seasonal influenza H3N2 vaccine
Source: PLoS Pathog. 2017 Oct 23;13(10):e1006682. doi: 10.1371/journal.ppat.1006682 (PMC5667890; doi:10.1371/journal.ppat.1006682)
Supplement: S2 Table — (PDF) [file ppat.1006682.s002.pdf]

Strain	Accession	Residue 194
HK14	EPI_ISL_270160	P
	EPI_ISL_233740	L
	EPI_ISL_202569	L
	EPI_ISL_198222	L
	EPI_ISL_195755	P
	EPI_ISL_189814	P
	EPI_ISL_176512	P
	EPI_ISL_165554	L
Sing16	EPI_ISL_239803	P
	EPI_ISL_225834	L
	EPI_ISL_275709	P
	EPI_ISL_257001	P
