# Supplementary material for: A structural explanation for the low effectiveness of the seasonal influenza H3N2 vaccine
Source: PLoS Pathog. 2017 Oct 23;13(10):e1006682. doi: 10.1371/journal.ppat.1006682 (PMC5667890; doi:10.1371/journal.ppat.1006682)
Supplement: S3 Table — (PDF) [file ppat.1006682.s003.pdf]

Data collection	Bris07 P194 Apo	Bris07 L194 Apo (Low Res)	Bris07 L194 Apo (High Res)
Beamline	APS 23ID-D	SSRL 12-2	SSRL 12-2
Wavelength (Å)	1.0332	0.9795	0.9795
Space group	H32	H32	H32
Unit cell parameters	a=b=100.5, c=383.3	a=b=100.6, c=382.6	a=b=100.6, c=384.2
Resolution (Å)	50-2.30 (2.37-2.30)[a]	50-2.35 (2.43-2.35)[a]	50-1.70 (1.76-1.70)[a]
Unique Reflections	34,068 (2,928)[a]	31,953 (3,148)[a]	81,614 (8,029)[a]
Redundancy	16.2 (9.0)[a]	15.5 (15.2)[a]	19.0 (18.7)[a]
Completeness (%)	99.6 (95.9)[a]	99.9 (100.0)[a]	100.0 (100.0)[a]
$\langle I/\sigma_I \rangle$	31.7 (1.6)[a]	34.1 (2.8)[a]	51.1 (3.1)[a]
R_{sym}[b]	0.10 (0.70)[a]	0.11 (0.84)[a]	0.09 (0.87)[a]
R_{pim}[b]	0.03 (0.22)[a]	0.03 (0.22)[a]	0.02 (0.21)[a]
$CC_{1/2}$[c]	1.00 (0.79)[a]	1.00 (0.93)[a]	1.00 (0.93)[a]
Z_a[d]	1	1	1
**Refinement statistics**			
Resolution (Å)	50-2.30	50-2.35	50-1.70
Reflections (work)	32,293	30,220	77,511
Reflections (test)	1,754	1,626	4,100
R_{cryst}(%)[e] / R_{free}(%)[f]	19.7 / 23.2	21.6 / 26.2	16.9 / 19.6
No. of atoms			
Protein	3,927	3,889	3,905
Water	164	127	492
Glycan	184	198	187
Average *B*-value (Å^2)			
Protein	59	65	35
Water	51	52	46
Glycan	98	99	57
Wilson *B*-value (Å^2)	40	43	22
**RMSD from ideal geometry**			
Bond length (Å)	0.012	0.010	0.012
Bond angle (°)	1.53	1.46	1.52
**Ramachandran statistics (%)**			
Favored	96.4	95.7	96.2
Outliers	0.0	0.0	0.2
**PDB code**	6AOP	6AOQ	6AOR

[a] Numbers in parentheses refer to the highest resolution shell.

[b] $R_{sym} = \Sigma_{hkl} \Sigma_i | I_{hkl,i} - \langle I_{hkl} \rangle | / \Sigma_{hkl} \Sigma_i I_{hkl,i}$ and $R_{pim} = \Sigma_{hkl} (1/(n-1))^{1/2} \Sigma_i | I_{hkl,i} - \langle I_{hkl} \rangle | / \Sigma_{hkl} \Sigma_i I_{hkl,i}$, where $I_{hkl,i}$ is the scaled intensity of the ith measurement of reflection h, k, l, $\langle I_{hkl} \rangle$ is the average intensity for that reflection, and *n* is the redundancy.

[c] $CC_{1/2}$ = Pearson correlation coefficient between two random half datasets.

[d] Z_a is the number of HA protomers per crystallographic asymmetric unit.

[e] $R_{cryst} = \Sigma_{hkl} | F_o - F_c | / \Sigma_{hkl} | F_o |$ x 100, where F_o and F_c are the observed and calculated structure factors, respectively.

[f] R_{free} was calculated as for R_{cryst}, but on a test set comprising 5% of the data excluded from refinement.
