# Supplementary material for: A structural explanation for the low effectiveness of the seasonal influenza H3N2 vaccine
Source: PLoS Pathog. 2017 Oct 23;13(10):e1006682. doi: 10.1371/journal.ppat.1006682 (PMC5667890; doi:10.1371/journal.ppat.1006682)
Supplement: S4 Table — (PDF) [file ppat.1006682.s004.pdf]

Data collection	Bris07 P194 3'SLNLN	Bris07 P194 6'SLNLN	Bris07 L194 3'SLNLN	Bris07 L194 6'SLNLN
Beamline	SSRL 12-2	SSRL 12-2	SSRL 12-2	SSRL 12-2
Wavelength (Å)	0.9795	0.9795	0.9795	0.9795
Space group	H32	H32	H32	H32
Unit cell parameters	a=b=100.4, c=383.3	a=b=100.3, c=384.3	a=b=100.6, c=383.1	a=b=100.7, c=385.1
Resolution (Å)	50-2.30 (2.37-2.30)[a]	50-1.95 (2.01-1.95)[a]	50-1.75 (1.81-1.75)[a]	50-1.75 (1.81-1.75)[a]
Unique Reflections	32,861 (2,878)[a]	55,042 (4,982)[a]	75,864 (7,484)[a]	75,889 (7,485)[a]
Redundancy	6.9 (6.6)[a]	9.1 (8.7)[a]	11.1 (9.8)[a]	18.5 (16.0)[a]
Completeness (%)	97.2 (96.4)[a]	99.2 (99.7)[a]	99.9 (99.9)[a]	99.8 (99.7)[a]
$<I/\sigma_I>$	22.6 (1.6)[a]	44.6 (3.4)[a]	43.0 (2.2)[a]	55.4 (2.7)[a]
R_{sym}[b]	0.12 (0.86)[a]	0.09 (0.78)[a]	0.07 (0.90)[a]	0.09 (0.92)[a]
R_{pim}[b]	0.05 (0.34)[a]	0.03 (0.28)[a]	0.02 (0.30)[a]	0.02 (0.23)[a]
$CC_{1/2}$[c]	1.00 (0.82)[a]	1.00 (0.91)[a]	1.00 (0.88)[a]	1.00 (0.94)[a]
Z_a[d]	1	1	1	1
**Refinement statistics**				
Resolution (Å)	50-2.30	50-1.95	50-1.75	50-1.75
Reflections (work)	30,895	52,423	72,214	72,000
Reflections (test)	1,609	2,651	3,641	3,862
R_{cryst}(%)[e] / R_{free}(%)[f]	20.6 / 25.2	18.3 / 20.3	16.8 / 19.0	17.4 / 19.6
No. of atoms				
Protein	3,897	3,902	3,910	3,908
Water	131	326	453	462
Glycan	173	173	198	184
Ligand[g]	46	32	21	71
Average *B*-value (Å^2)				
Protein	55	41	36	38
Water	51	48	46	46
Glycan	92	71	60	64
Ligand[g]	92	68	101	75
Wilson *B*-value (Å^2)	42	31	28	24
**RMSD from ideal geometry**				
Bond length (Å)	0.011	0.012	0.012	0.012
Bond angle (°)	1.54	1.55	1.54	1.56
**Ramachandran statistics (%)**				
Favored	96.2	96.0	96.2	96.2
Outliers	0.0	0.2	0.0	0.2
**PDB code**	6AOS	6AOT	6AOU	6AOV

[a] Numbers in parentheses refer to the highest resolution shell.

[b] $R_{sym} = \Sigma_{hkl} \Sigma_i | I_{hkl,i} - <I_{hkl}> | / \Sigma_{hkl} \Sigma_i I_{hkl,i}$ and $R_{pim} = \Sigma_{hkl} (1/(n-1))^{1/2} \Sigma_i | I_{hkl,i} - <I_{hkl}> | / \Sigma_{hkl} \Sigma_i I_{hkl,i}$, where $I_{hkl,i}$ is the scaled intensity of the ith measurement of reflection h, k, l, $<I_{hkl}>$ is the average intensity for that reflection, and *n* is the redundancy.

[c] $CC_{1/2}$ = Pearson correlation coefficient between two random half datasets.

[d] Z_a is the number of HA protomers per crystallographic asymmetric unit.

[e] $R_{cryst} = \Sigma_{hkl} | F_o - F_c | / \Sigma_{hkl} | F_o |$ x 100, where F_o and F_c are the observed and calculated structure factors, respectively.

[f] R_{free} was calculated as for R_{cryst}, but on a test set comprising 5% of the data excluded from refinement.

[g] Either 3'SLNLN or 6'SLNLN.
