# Supplementary material for: A structural explanation for the low effectiveness of the seasonal influenza H3N2 vaccine
Source: PLoS Pathog. 2017 Oct 23;13(10):e1006682. doi: 10.1371/journal.ppat.1006682 (PMC5667890; doi:10.1371/journal.ppat.1006682)
Supplement: S5 Table — (PDF) [file ppat.1006682.s005.pdf]

HA Protein	Crystallization Condition
Bris07 P194	0.1 M CAPS pH 10.5 and 29% PEG 400
Bris07 L194 (high resolution)	0.1 M bicine pH 9 and 41% 2-Methyl-2,4-pentanediol
Bris07 L194 (low resolution)	0.1 M CAPS pH 10.5 and 29% PEG 400
