# Supplementary figures and images for: A structural explanation for the low effectiveness of the seasonal influenza H3N2 vaccine
Source: PLoS Pathog. 2017 Oct 23;13(10):e1006682. doi: 10.1371/journal.ppat.1006682 (PMC5667890; doi:10.1371/journal.ppat.1006682)

**Bris07 P194**

**Bris07 L194**

**190-helix**

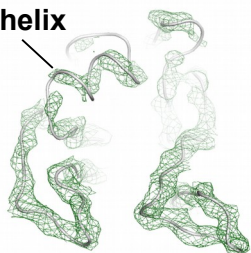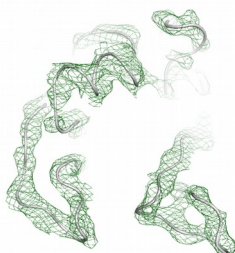

**1.2  $\sigma$**

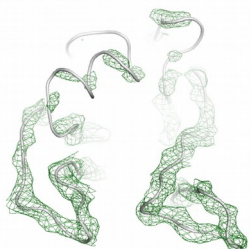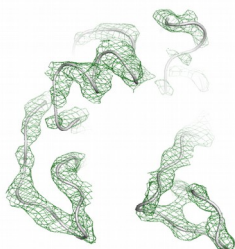

**1.4  $\sigma$**

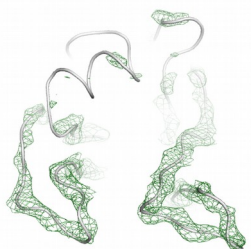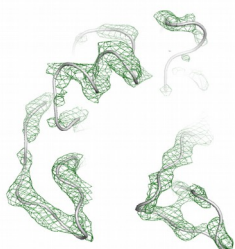

**1.6  $\sigma$**

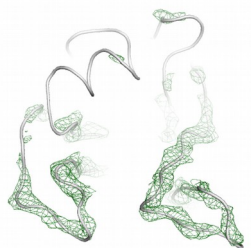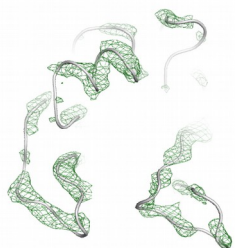

**1.8  $\sigma$**

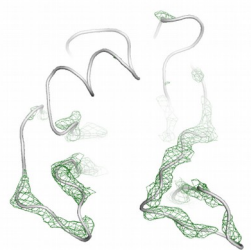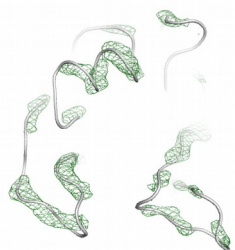

**2.0  $\sigma$**

Supplement: S1 Fig — Final 2Fo-Fc electron density maps for the backbone of the receptor binding sites (grey sticks) are represented in a green mesh and contoured at the indicated σ levels to illustrate differences in electron density levels between Bris07 P194 and L194 HAs. (PDF) [file ppat.1006682.s006.pdf]

**A****Bris07 P194**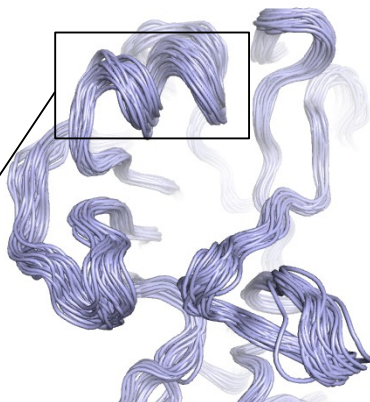**Bris07 L194**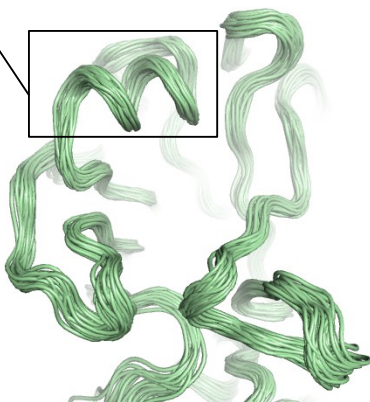**B**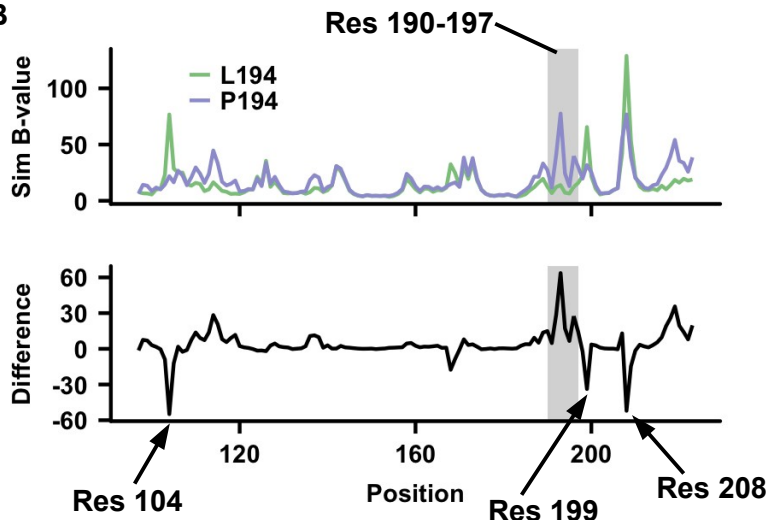**C**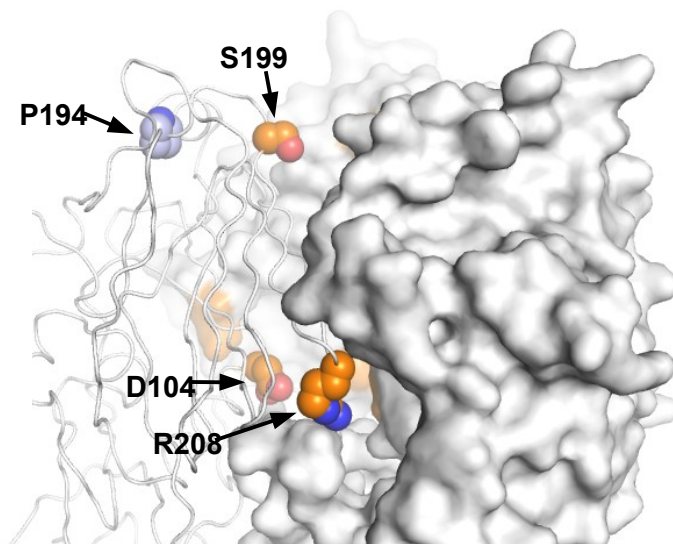

Supplement: S2 Fig — (A) 50 random frames from the 500 ns molecular dynamics simulation for Bris07 P194 (blue) and L194 (green) are aligned. (B) The simulated B-values of Cα atoms in Bris07 P194 and L194 are compared. The bottom panel shows their difference: (simulated B-values of Cα atoms in P194)–(simulated B-values of Cα atoms in L194). Positions corresponding to the residues (Res) of interest are shaded in grey. The positions of three sharp inverse peaks are indicated. Of note, these inverse peaks were not observed in normalized B-value analysis (Fig 2C). (C) Those residues (orange) corresponding to the inverse peaks in panel B, along with P194 (blue), are shown on the HA trimer structure. One protomer of the HA trimer is shown in cartoon representation and the other two protomers are shown in surface representation. Those three residues (D104, S199, and R208) are either involved in or close to the interface between protomers. Therefore, we rationalized that these inverse peaks in panel B represent an artifact from molecular dynamics simulation due to the usage of a monomeric instead of trimeric structure. (PDF) [file ppat.1006682.s007.pdf]

## Residues 214 to 218

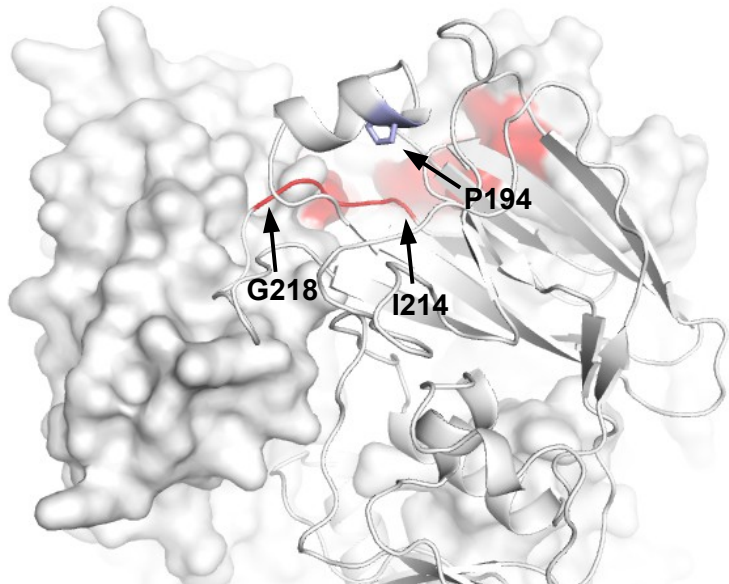

Supplement: S3 Fig — The locations of residues 214–218, which have a higher normalized B-value in Bris07 P194 as compared to Bris07 L194, are highlighted in red in all three protomers of the HA trimer. One protomer is shown in cartoon representation and the other two protomers are shown in surface representation. P194 is shown as stick representation and colored blue. (PDF) [file ppat.1006682.s008.pdf]

Switz13 WT Switz13 L194P

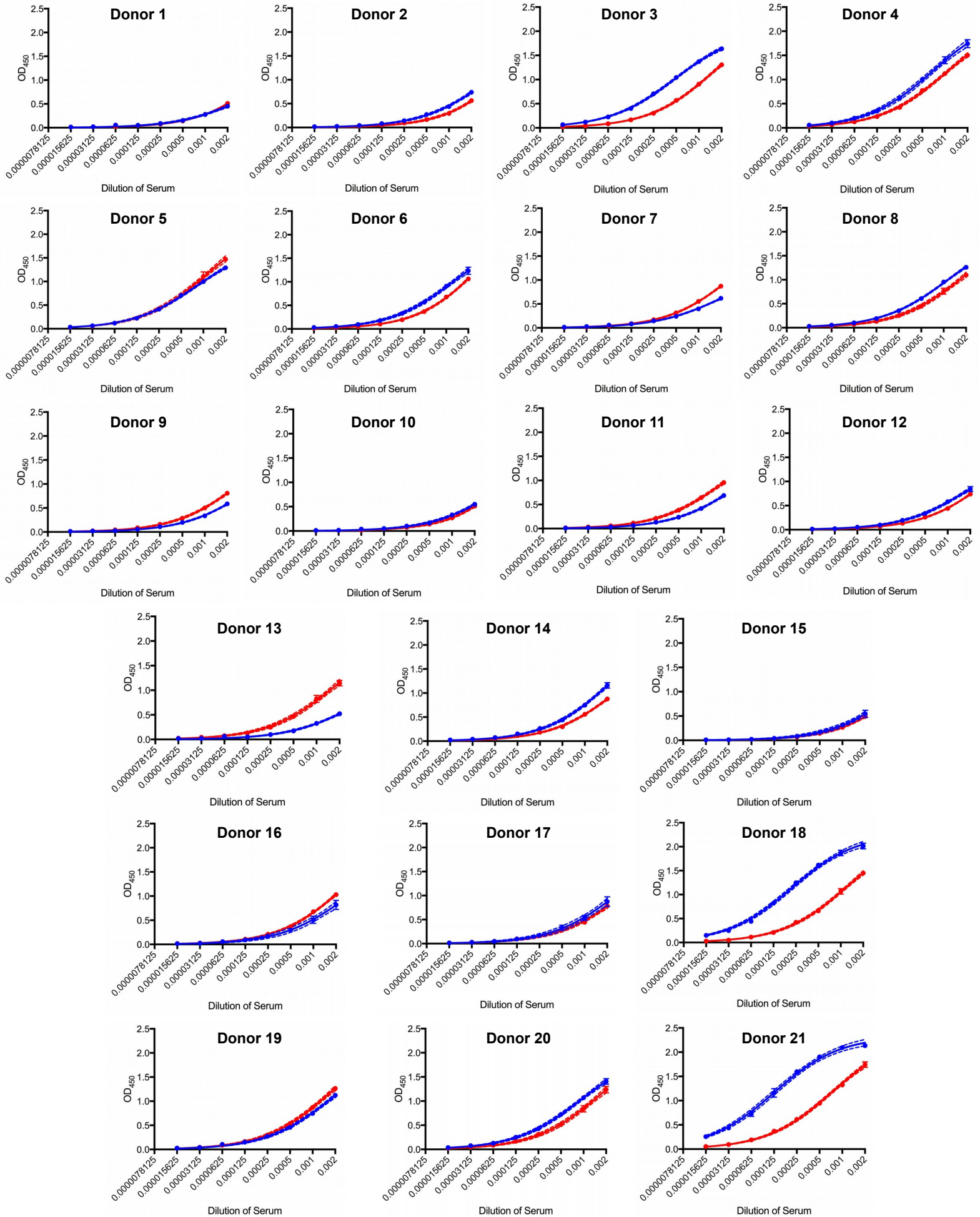

Supplement: S4 Fig — Error bars for individual points represent the standard error of the mean (SEM), while dashed lines represent the 95% confidence interval of the one-site specific binding curve fit to the ELISA data (N = 3 experimental replicates). (PDF) [file ppat.1006682.s009.pdf]

Switz13 WT Switz13 L194P

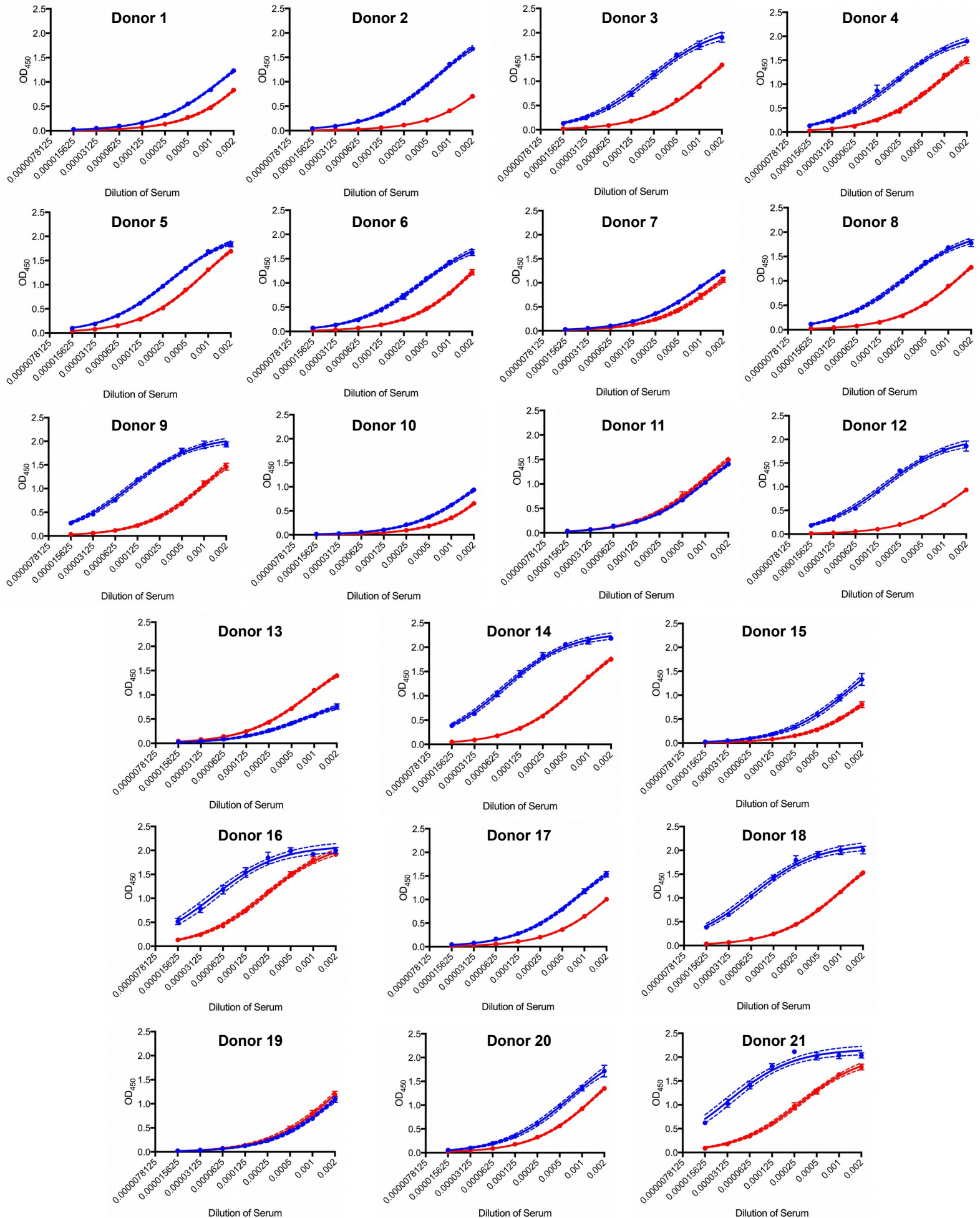

Supplement: S5 Fig — Error bars for individual points represent the standard error of the mean (SEM), while dashed lines represent the 95% confidence interval of the one-site specific binding curve fit to the ELISA data (N = 3 experimental replicates). (PDF) [file ppat.1006682.s010.pdf]

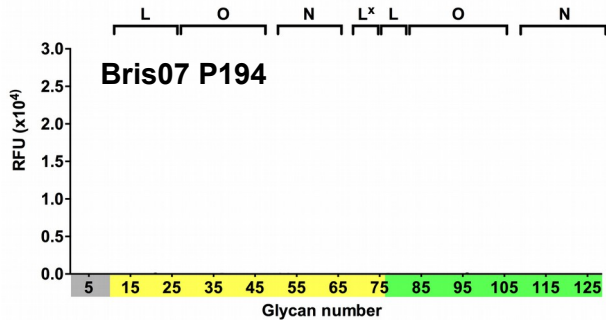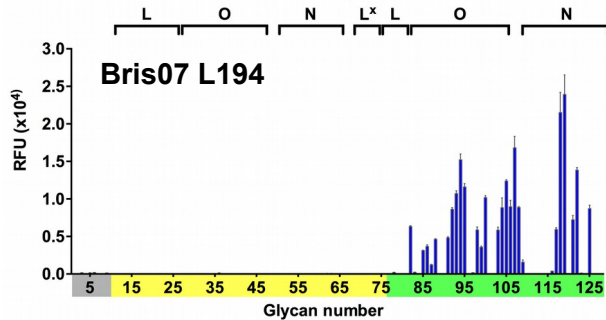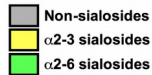

Supplement: S6 Fig — 293S-expressed recombinant HA was purified and analyzed by the sialoside glycan array. (PDF) [file ppat.1006682.s011.pdf]

**2Fo-Fc map**

**Omit (Fo-Fc) map**

**3'SLNLN  
in P194**

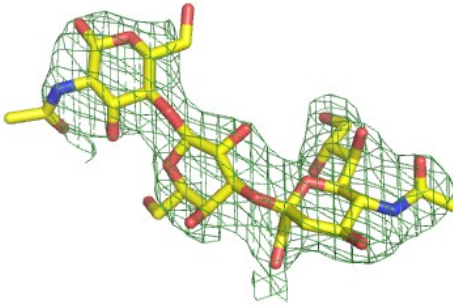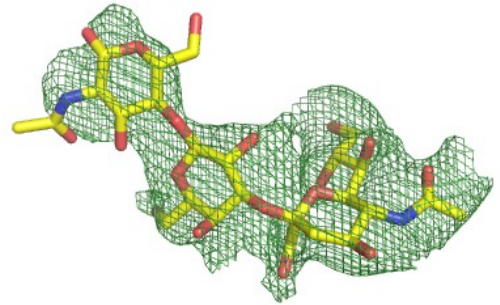

**6'SLNLN  
in P194**

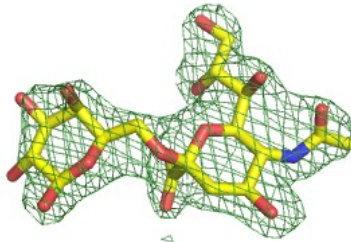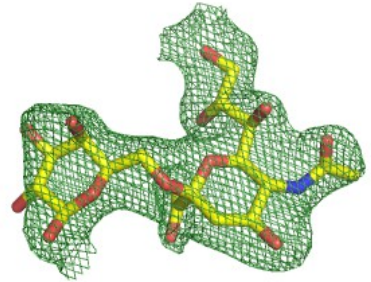

**3'SLNLN  
in L194**

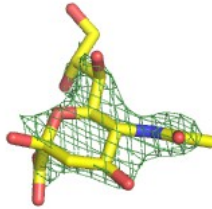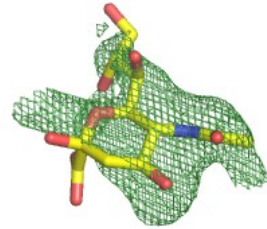

**6'SLNLN  
in L194**

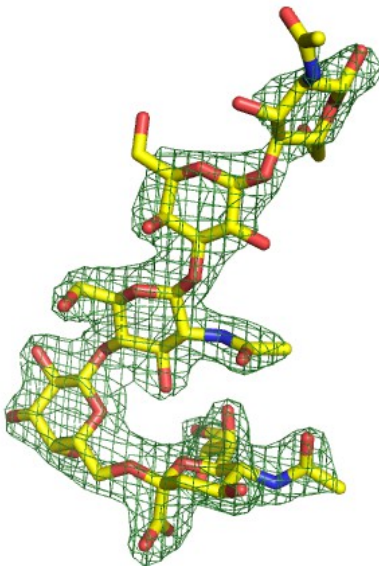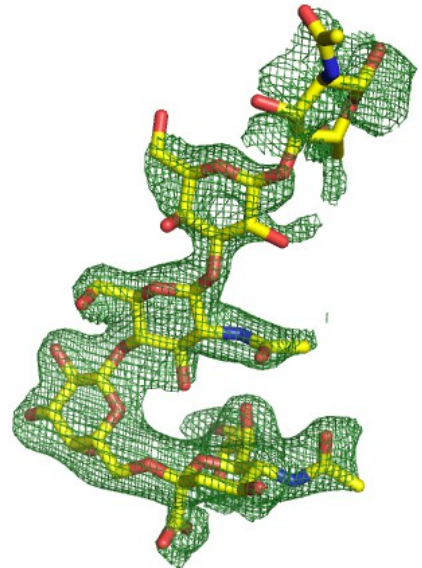

Supplement: S7 Fig — Final 2Fo-Fc electron density maps for the glycan receptor analogs (yellow sticks) are represented in a green mesh and contoured at 0.9 σ (left). Omit (Fo-Fc) electron density maps for the glycan receptor analogs are represented in a green mesh and contoured at 2.0 σ (right). (PDF) [file ppat.1006682.s012.pdf]

A/turkey/Turkey/1/2005 H5 (PDB 4BH0)

A/Vietnam/1194/2004 H5 (PDB 4BGX)

Bris07 P194

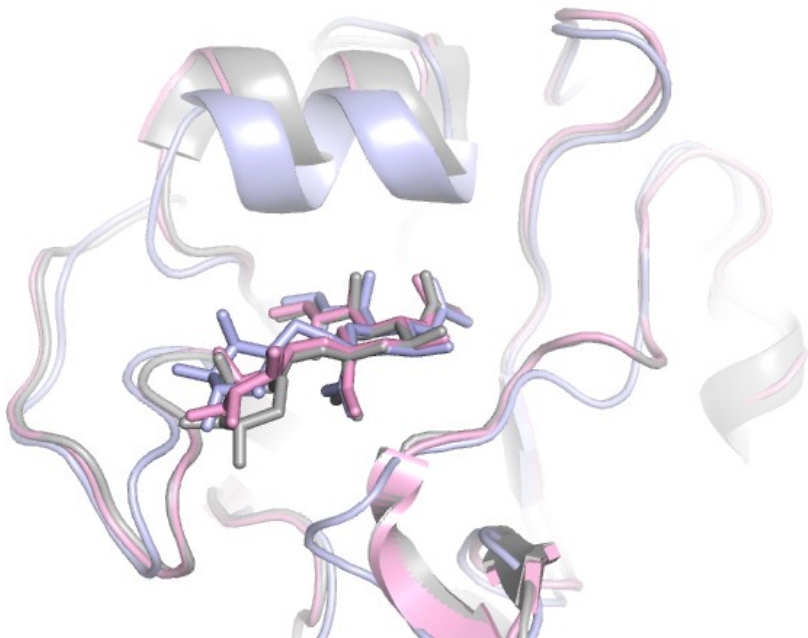

Supplement: S8 Fig — The structures of A/turkey/Turkey/1/2005 H5 HA in complex with 6'SLN (PDB 4BH0 [29]) and A/Vietnam/1194/2004 H5 HA in complex with 6'SLN (PDB 4BGX [29]) are aligned to the structure of Bris07 P194 in complex with 6'SLNLN. HAs are shown in cartoon representations. The first two monosaccharide moieties of the human receptor analogs (6'SLN and 6'SLNLN) are shown in stick representations. (PDF) [file ppat.1006682.s013.pdf]

**A/Hong Kong/1/1968 H3 (PDB 2YPG)**

**Bris07 L194**

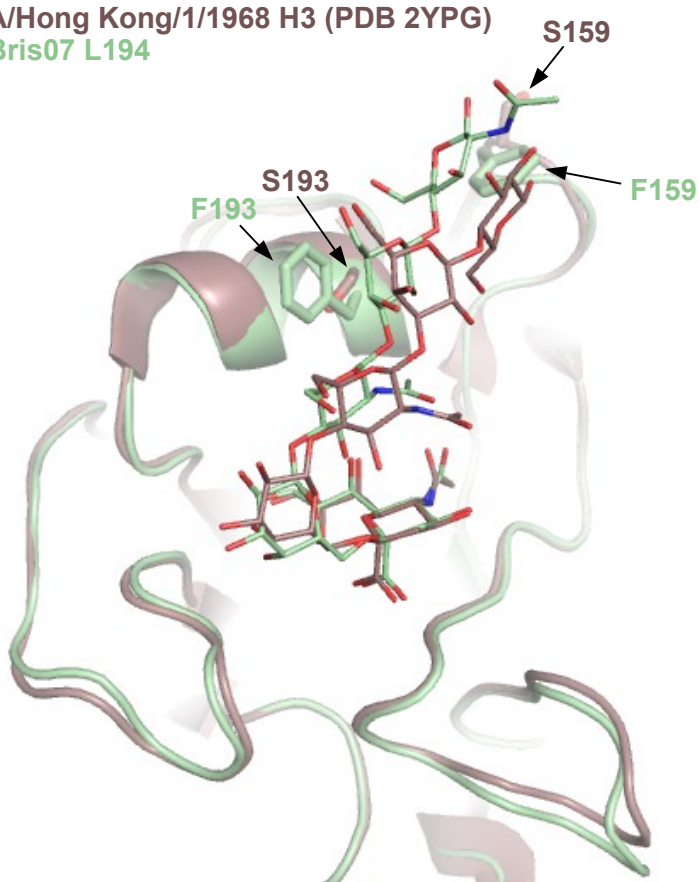

Supplement: S9 Fig — The structure of Bris07 L194 in complex with human receptor analog 6'SLNLN is aligned with the structure of HK68 HA in complex with human receptor analog sialylneolacto-N-tetraose c (PDB 2YPG [32]) based on the receptor-binding subdomain (residues 117–265 [58]). (PDF) [file ppat.1006682.s014.pdf]

**A**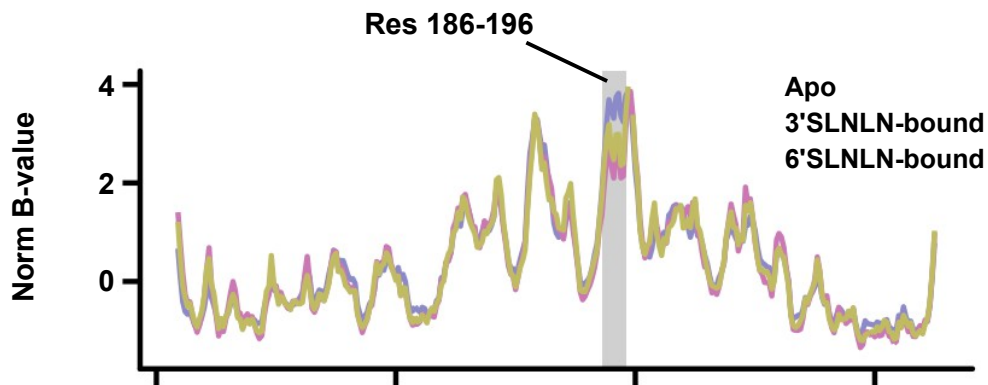**B**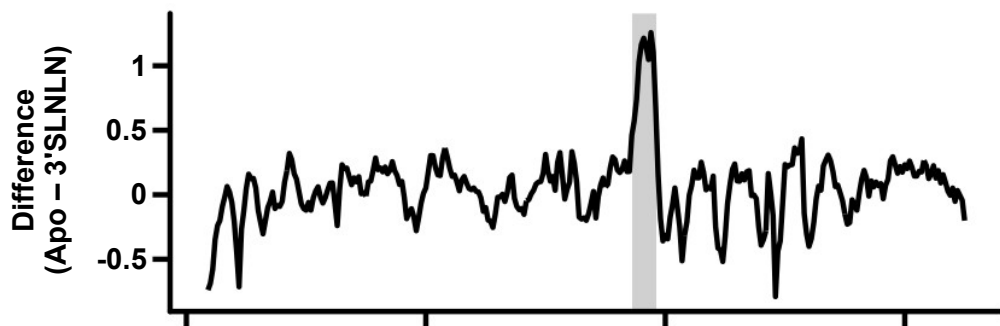**C**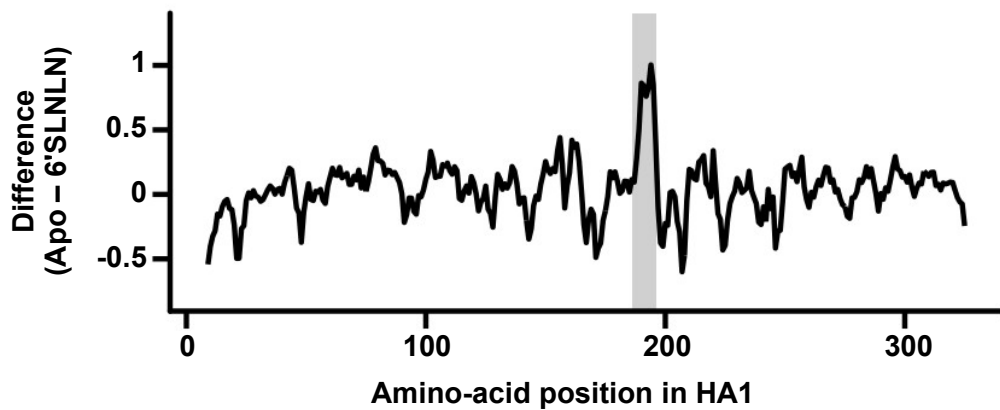

Supplement: S10 Fig — (A) The normalized B-values of Cα atoms in Bris07 P194 apo form, 3'SLNLN-bound form, 6'SLNLN-bound form are compared. (B) The difference between the normalized B-value in apo and 3'SLNLN-bound forms is shown. Difference = (normalized B-values of Cα atoms in apo form)–(normalized B-values of Cα atoms in 3'SLNLN). (C) The difference between the normalized B-value in apo and 6'SLNLN-bound forms is shown. Difference = (normalized B-values of Cα atoms in apo form)–(normalized B-values of Cα atoms in 6'SLNLN). The amino-acid position is plotted along the x-axis. Positions corresponding to the residues (Res) of interest are shaded in grey. (PDF) [file ppat.1006682.s015.pdf]
