# Supplementary material for: A structural explanation for the low effectiveness of the seasonal influenza H3N2 vaccine
Source: PLoS Pathog. 2017 Oct 23;13(10):e1006682. doi: 10.1371/journal.ppat.1006682 (PMC5667890; doi:10.1371/journal.ppat.1006682)
Supplement: S11 Fig — The name and structure of each compound on the glycan array are shown. (PDF) [file ppat.1006682.s016.pdf]

Glycan #	Common Name	Structure
1	Galβ(1-4)GlcNAcβ-ethyl-NH2	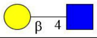
2	Galβ(1-4)GlcNAcβ(1-3)Galβ(1-3)GalNAcα-Thr-NH2	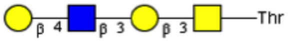
3	Galβ(1-4)GlcNAcβ(1-6)[Galβ(1-3)]-GalNAcα-Thr-NH2	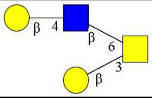
4	Galβ(1-4)GlcNAcβ(1-3)GalNAcα-Thr-NH2	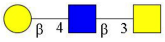
5	Galβ(1-4)GlcNAcβ(1-3)[Galβ(1-4)GlcNAcβ(1-6)]-GalNAcα-Thr-NH2	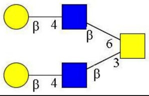
6	Galβ(1-4)GlcNAcβ(1-6)GalNAcα-Thr-NH2	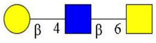
7	Galβ(1-4)GlcNAcβ(1-2)Manα (1-3)[Galβ(1-4)GlcNAcβ(1-2)Manα(1-6)]-Manβ(1-4)GlcNAcβ(1-4)GlcNAcβ-Asn-NH2	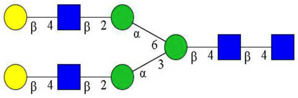
8	Galβ(1-4)GlcNAcβ(1-2)Manα(1-3)[Galβ(1-4)GlcNAcβ(1-2)Manα(1-6)]-Manβ(1-4)GlcNAcβ(1-4)[Fucα(1-6)]-GlcNAcβ-Asn-Ser-Thr-NH2	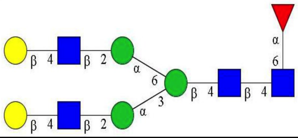
9	Galβ(1-4)GlcNAcβ(1-2)Manα(1-3){Galβ(1-4)GlcNAcβ(1-2)[Galβ(1-4)GlcNAcβ(1-2)]-Manα(1-6)}-Manβ(1-4)GlcNAcβ(1-4)GlcNAcβ-Asn-Lys-NH2	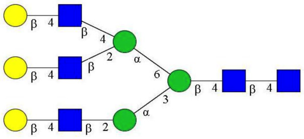
10	Galβ(1-4)GlcNAcβ(1-2)Manα(1-3){Galβ(1-4)GlcNAcβ(1-2)[Galβ(1-4)GlcNAcβ(1-2)]-Manα(1-6)}-Manβ(1-4)GlcNAcβ(1-4)[Fucα(1-6)]-GlcNAcβ-(Lys-Val-Ala)Asn-Lys-Thr-NH2	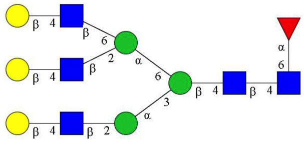
11	NeuAcα(2-3)Galβ(1-4)6-O-sulfo-GlcNAcβ-propyl-NH2	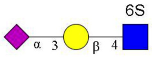
12	NeuAcα(2-3)Galβ(1-4)[Fucα(1-3)]-6-O-sulfo-GlcNAcβ-propyl-NH2	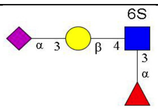
13	NeuAcα(2-3)6-O-sulfo-Galβ(1-4)GlcNAcβ-ethyl-NH2	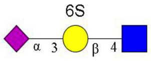
14	NeuAcα(2-3)6-O-sulfo-Galβ(1-4)[Fucα(1-3)]-GlcNAcβ-propyl-NH2	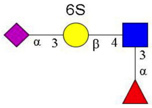
15	NeuAcα(2-3)Galβ(1-3)6-O-sulfo-GlcNAcβ-propyl-NH2	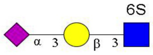
16	NeuAcα(2-3)Galβ(1-4)Glcβ-ethyl-NH2	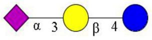
17	NeuAcα(2-3)Galβ(1-4)GlcNAcβ-ethyl-NH2	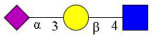
18	NeuAcα(2-3)Galβ(1-4)GlcNAcβ(1-3)Galβ(1-4)GlcNAcβ-ethyl-NH2	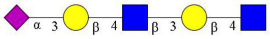
19	NeuAcα(2-3)Galβ(1-4)GlcNAcβ(1-3)Galβ(1-4)GlcNAcβ(1-3)Galβ(1-4)GlcNAcβ-ethyl-NH2	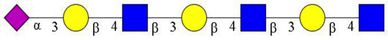

Glycan #	Common Name	Structure
20	NeuAcα(2-3)GalNAcβ(1-4)GlcNAcβ-ethyl-NH2	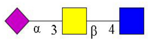
21	NeuAcα(2-3)Galβ(1-3)GlcNAcβ-ethyl-NH2	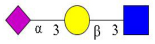
22	NeuAcα(2-3)Galβ(1-3)GlcNAcβ(1-3)Galβ(1-4)GlcNAcβ-ethyl-NH2	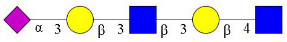
23	NeuAcα(2-3)Galβ(1-3)GlcNAcβ(1-3)Galβ(1-3)GlcNAcβ-ethyl-NH2	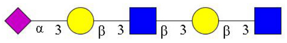
24	NeuAcα(2-3)Galβ(1-3)GalNAcβ(1-3)Galα(1-4)Galβ(1-4)Glcβ-ethyl-NH2	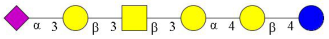
25	NeuAcα(2-3)Galβ(1-3)GalNAcα-Thr-NH2	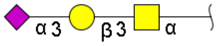
26	NeuAcα(2-3)Galβ(1-4)GlcNAcβ(1-3)Galβ(1-3)GalNAcα-Thr-NH2	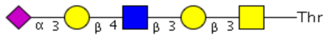
27	NeuAcα(2-3)Galβ(1-4)GlcNAcβ(1-3)Galβ(1-4)GlcNAcβ(1-3)Galβ(1-3)GalNAcα-Thr-NH2	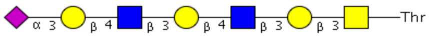
28	NeuAcα(2-3)Galβ(1-4)GlcNAcβ(1-3)Galβ(1-4)GlcNAcβ(1-3)Galβ(1-4)GlcNAcβ(1-3)Galβ(1-3)GalNAcα-Thr-NH2	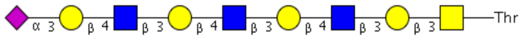
29	NeuAcα(2-3)Galβ(1-4)GlcNAcβ(1-3)Galβ(1-4)GlcNAcβ(1-3)Galβ(1-4)GlcNAcβ(1-3)Galβ(1-4)GlcNAcβ(1-3)Galβ(1-3)GalNAcα-Thr-NH2	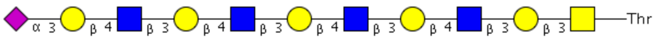
30	NeuAcα(2-3)Galβ(1-4)GlcNAcβ(1-3)Galβ(1-4)GlcNAcβ(1-3)Galβ(1-4)GlcNAcβ(1-3)Galβ(1-4)GlcNAcβ(1-3)Galβ(1-4)GlcNAcβ(1-3)Galβ(1-3)GalNAcα-Thr-NH2	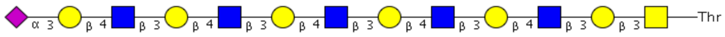
31	NeuAcα(2-3)Galβ(1-4)GlcNAcβ(1-6)[Galβ(1-3)]-GalNAcα-Thr-NH2	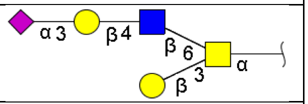
32	NeuAcα(2-3)Galβ(1-4)GlcNAcβ(1-3)Galβ(1-4)GlcNAcβ(1-6)[Galβ(1-3)]-GalNAcα-Thr-NH2	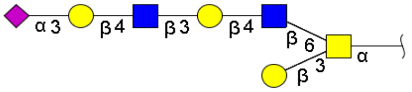
33	NeuAcα(2-3)Galβ(1-4)GlcNAcβ(1-3)Galβ(1-4)GlcNAcβ(1-3)Galβ(1-4)GlcNAcβ(1-6)[Galβ(1-3)]-GalNAcα-Thr-NH2	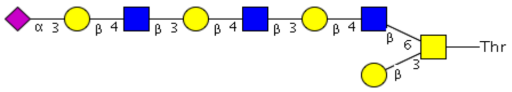
34	NeuAcα(2-3)Galβ(1-4)GlcNAcβ(1-3)Galβ(1-4)GlcNAcβ(1-3)Galβ(1-4)GlcNAcβ(1-3)Galβ(1-4)GlcNAcβ(1-6)[Galβ(1-3)]-GalNAcα-Thr-NH2	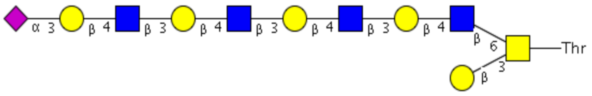
35	NeuAcα(2-3)Galβ(1-4)GlcNAcβ(1-3)Galβ(1-4)GlcNAcβ(1-3)Galβ(1-4)GlcNAcβ(1-3)Galβ(1-4)GlcNAcβ(1-3)Galβ(1-4)GlcNAcβ(1-6)[Galβ(1-3)]-GalNAcα-Thr-NH2	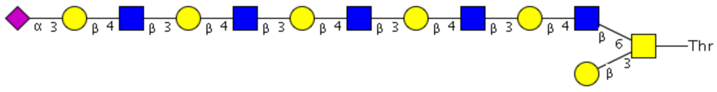
36	NeuAcα(2-3)Galβ(1-4)GlcNAcβ(1-3)Galβ(1-4)GlcNAcβ(1-3)Galβ(1-4)GlcNAcβ(1-3)Galβ(1-4)GlcNAcβ(1-6)[NeuAcα(2-3)Galβ(1-4)GlcNAcβ(1-3)Galβ(1-4)GlcNAcβ(1-3)Galβ(1-4)GlcNAcβ(1-3)Galβ(1-3)]-GalNAcα-Thr-NH2	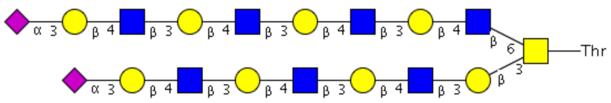

Glycan #	Common Name	Structure
**37**	NeuAcα(2-3)Galβ(1-4)GlcNAcβ(1-3)Galβ(1-4)GlcNAcβ(1-3)Galβ(1-4)GlcNAcβ(1-3)Galβ(1-4)GlcNAcβ(1-3)Galβ(1-4)GlcNAcβ(1-6)[NeuAcα(2-3)Galβ(1-4)GlcNAcβ(1-3)Galβ(1-4)GlcNAcβ(1-3)Galβ(1-4)GlcNAcβ(1-3)Galβ(1-4)GlcNAcβ(1-3)Galβ(1-3)]-GalNAcα-Thr-NH2	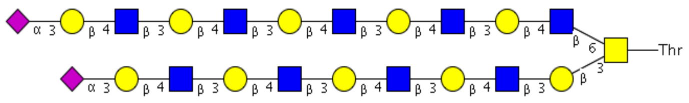
**38**	NeuAcα(2-3)Galβ(1-4)GlcNAcβ(1-3)GalNAcα-Thr-NH2	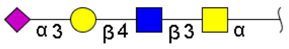
**39**	NeuAcα(2-3)Galβ(1-4)GlcNAcβ(1-3)Galβ(1-4)GlcNAcβ(1-3)GalNAcα-Thr-NH2	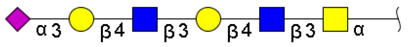
**40**	NeuAcα(2-3)Galβ(1-4)GlcNAcβ(1-3)Galβ(1-4)GlcNAcβ(1-3)Galβ(1-4)GlcNAcβ(1-3)GalNAcα-Thr-NH2	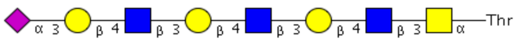
**41**	NeuAcα(2-3)Galβ(1-4)GlcNAcβ(1-3)Galβ(1-4)GlcNAcβ(1-3)Galβ(1-4)GlcNAcβ(1-3)Galβ(1-4)GlcNAcβ(1-3)GalNAcα-Thr-NH2	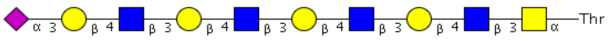
**42**	NeuAcα(2-3)Galβ(1-4)GlcNAcβ(1-3)Galβ(1-4)GlcNAcβ(1-3)Galβ(1-4)GlcNAcβ(1-3)Galβ(1-4)GlcNAcβ(1-3)Galβ(1-4)GlcNAcβ(1-3)GalNAcα-Thr-NH2	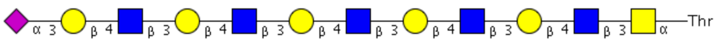
**43**	NeuAcα(2-3)Galβ(1-4)GlcNAcβ(1-3)[NeuAcα(2-3)Galβ(1-4)GlcNAcβ(1-6)]-GalNAcα-Thr-NH2	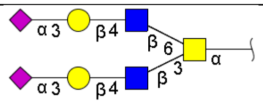
**44**	NeuAcα(2-3)Galβ(1-4)GlcNAcβ(1-3)Galβ(1-4)GlcNAcβ(1-3)[NeuAcα(2-3)Galβ(1-4)GlcNAcβ(1-3)Galβ(1-4)GlcNAcβ(1-6)]-GalNAcα-Thr-NH2	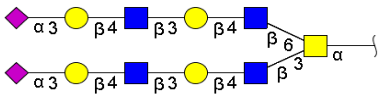
**45**	NeuAcα(2-3)Galβ(1-4)GlcNAcβ(1-3)Galβ(1-4)GlcNAcβ(1-3)Galβ(1-4)GlcNAcβ(1-3)[NeuAcα(2-3)Galβ(1-4)GlcNAcβ(1-3)Galβ(1-4)GlcNAcβ(1-3)Galβ(1-4)GlcNAcβ(1-6)]-GalNAcα-Thr-NH2	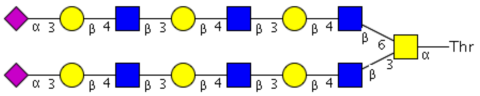
**46**	NeuAcα(2-3)Galβ(1-4)GlcNAcβ(1-3)Galβ(1-4)GlcNAcβ(1-3)Galβ(1-4)GlcNAcβ(1-3)Galβ(1-4)GlcNAcβ(1-3)[NeuAcα(2-3)Galβ(1-4)GlcNAcβ(1-3)Galβ(1-4)GlcNAcβ(1-3)Galβ(1-4)GlcNAcβ(1-3)Galβ(1-4)GlcNAcβ(1-6)]-GalNAcα-Thr-NH2	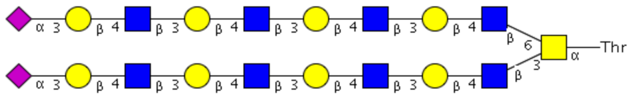
**47**	NeuAcα(2-3)Galβ(1-4)GlcNAcβ(1-3)Galβ(1-4)GlcNAcβ(1-3)Galβ(1-4)GlcNAcβ(1-3)Galβ(1-4)GlcNAcβ(1-3)Galβ(1-4)GlcNAcβ(1-3)[NeuAcα(2-3)Galβ(1-4)GlcNAcβ(1-3)Galβ(1-4)GlcNAcβ(1-3)Galβ(1-4)GlcNAcβ(1-3)Galβ(1-4)GlcNAcβ(1-3)Galβ(1-4)GlcNAcβ(1-6)]-GalNAcα-Thr-NH2	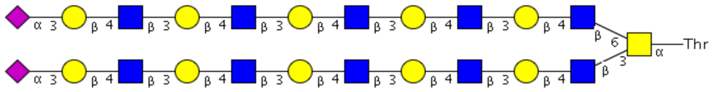
**48**	NeuAcα(2-3)Galβ(1-4)GlcNAcβ(1-3)Galβ(1-4)GlcNAcβ(1-3)Galβ(1-4)GlcNAcβ(1-3)Galβ(1-4)GlcNAcβ(1-6)GalNAcα-Thr-NH2	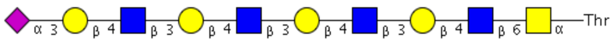

Glycan #	Common Name	Structure
**49**	NeuAcα(2-3)Galβ(1-4)GlcNAcβ(1-3)Galβ(1-4)GlcNAcβ(1-3)Galβ(1-4)GlcNAcβ(1-3)Galβ(1-4)GlcNAcβ(1-3)Galβ(1-4)GlcNAcβ(1-6)GalNAcα-Thr-NH2	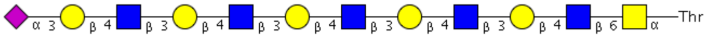
**50**	NeuAcα(2-3)Galβ(1-3)GlcNAcβ(1-3)Galβ(1-4)GlcNAcβ(1-6)[NeuAcα(2-3)Galβ(1-3)GlcNAcβ(1-3)] Galβ(1-4)GlcNAcβ-ethyl-NH2	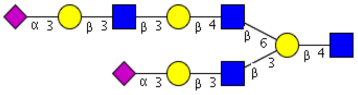
**51**	NeuAcα(2-3)Galβ(1-4)GlcNAcβ(1-3)Galβ(1-4)GlcNAcβ(1-3)Galβ(1-4)GlcNAcβ(1-6)[NeuAcα(2-3)Galβ(1-4)GlcNAcβ(1-3)Galβ(1-4)GlcNAcβ(1-3)] Galβ(1-4)GlcNAcβ-ethyl-NH2	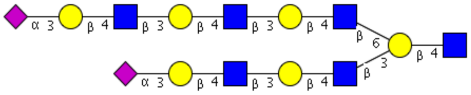
**52**	NeuAcα(2-3)Galβ(1-4)GlcNAcβ(1-2)Manα(1-3)[NeuAcα(2-3)Galβ(1-4)GlcNAcβ(1-2)Manα(1-6)]-Manβ(1-4)GlcNAcβ(1-4)GlcNAcβ-Asn-NH2	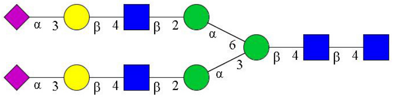
**53**	NeuAcα(2-3)Galβ(1-4)GlcNAcβ(1-3)Galβ(1-4)GlcNAcβ(1-2)Manα(1-3)[NeuAcα(2-3)Galβ(1-4)GlcNAcβ(1-3)Galβ(1-4)GlcNAcβ(1-2)Manα(1-6)]-Manβ(1-4)GlcNAcβ(1-4)GlcNAcβ-Asn-NH2	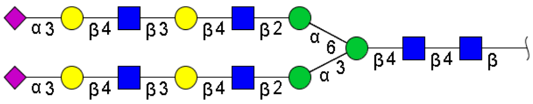
**54**	NeuAcα(2-3)Galβ(1-4)GlcNAcβ(1-3)Galβ(1-4)GlcNAcβ(1-3)Galβ(1-4)GlcNAcβ(1-2)Manα(1-3)[NeuAcα(2-3)Galβ(1-4)GlcNAcβ(1-3)Galβ(1-4)GlcNAcβ(1-3)Galβ(1-4)GlcNAcβ(1-2)Manα(1-6)]-Manβ(1-4)GlcNAcβ(1-4)GlcNAcβ-Asn-NH2	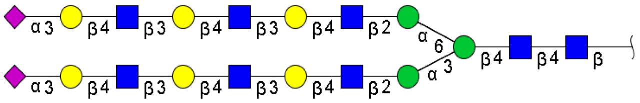
**55**	NeuAcα(2-3)Galβ(1-4)GlcNAcβ(1-3)Galβ(1-4)GlcNAcβ(1-2)Manα(1-3)[NeuAcα(2-3)Galβ(1-4)GlcNAcβ(1-3)Galβ(1-4)GlcNAcβ(1-2)Manα(1-6)]-Manβ(1-4)GlcNAcβ(1-4)GlcNAcβ-(Lys-Val-Ala)Asn-Lys-Thr-NH2	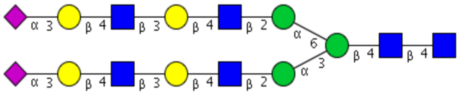
**56**	NeuAcα(2-3)Galβ(1-4)GlcNAcβ(1-3)Galβ(1-4)GlcNAcβ(1-3)Galβ(1-4)GlcNAcβ(1-2)Manα(1-3)[NeuAcα(2-3)Galβ(1-4)GlcNAcβ(1-3)Galβ(1-4)GlcNAcβ(1-3)Galβ(1-4)GlcNAcβ(1-2)Manα(1-6)]-Manβ(1-4)GlcNAcβ(1-4)GlcNAcβ-(Lys-Val-Ala)Asn-Lys-Thr-NH2	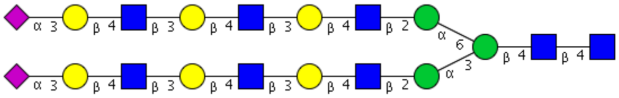
**57**	NeuAcα(2-3)Galβ(1-4)GlcNAcβ(1-3)Galβ(1-4)GlcNAcβ(1-3)Galβ(1-4)GlcNAcβ(1-3)Galβ(1-4)GlcNAcβ(1-2)Manα(1-3)[NeuAcα(2-3)Galβ(1-4)GlcNAcβ(1-3)Galβ(1-4)GlcNAcβ(1-3)Galβ(1-4)GlcNAcβ(1-3)Galβ(1-4)GlcNAcβ(1-2)Manα(1-6)]-Manβ(1-4)GlcNAcβ(1-4)GlcNAcβ-(Lys-Val-Ala)Asn-Lys-Thr-NH2	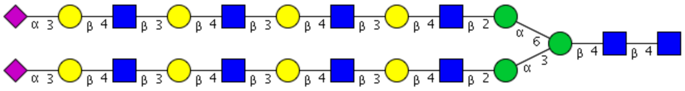

Glycan #	Common Name	Structure
**58**	NeuAcα(2-3)Galβ(1-4)GlcNAcβ(1-3)Galβ(1-4)GlcNAcβ(1-3)Galβ(1-4)GlcNAcβ(1-3)Galβ(1-4)GlcNAcβ(1-3)Galβ(1-4)GlcNAcβ(1-2)Manα(1-3)[NeuAcα(2-3)Galβ(1-4)GlcNAcβ(1-3)Galβ(1-4)GlcNAcβ(1-3)Galβ(1-4)GlcNAcβ(1-3)Galβ(1-4)GlcNAcβ(1-2)Manα(1-6)]-Manβ(1-4)GlcNAcβ(1-4)GlcNAcβ-(Lys-Val-Ala)Asn-Lys-Thr-NH2	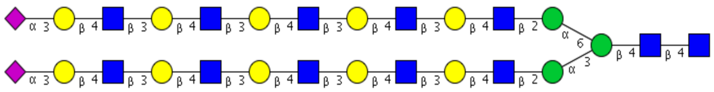
**59**	NeuAcα(2-3)Galβ(1-4)GlcNAcβ(1-3)Galβ(1-4)GlcNAcβ(1-3)Galβ(1-4)GlcNAcβ(1-2)Manα(1-3)[NeuAcα(2-3)Galβ(1-4)GlcNAcβ(1-3)Galβ(1-4)GlcNAcβ(1-3)Galβ(1-4)GlcNAcβ(1-2)Manα(1-6)]-Manβ(1-4)GlcNAcβ(1-4)[Fucα(1-6)]-GlcNAcβ-(Lys-Val-Ala)Asn-Lys-Thr-NH2	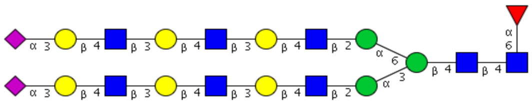
**60**	NeuAcα(2-3)Galβ(1-4)GlcNAcβ(1-3)Galβ(1-4)GlcNAcβ(1-3)Galβ(1-4)GlcNAcβ(1-3)Galβ(1-4)GlcNAcβ(1-2)Manα(1-3)[NeuAcα(2-3)Galβ(1-4)GlcNAcβ(1-3)Galβ(1-4)GlcNAcβ(1-3)Galβ(1-4)GlcNAcβ(1-3)Galβ(1-4)GlcNAcβ(1-2)Manα(1-6)]-Manβ(1-4)GlcNAcβ(1-4)[Fucα(1-6)]-GlcNAcβ-(Lys-Val-Ala)Asn-Lys-Thr-NH2	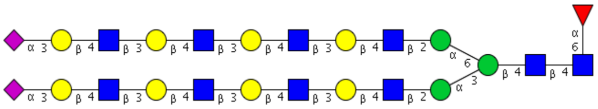
**61**	NeuAcα(2-3)Galβ(1-4)GlcNAcβ(1-3)Galβ(1-4)GlcNAcβ(1-2)Manα(1-3){NeuAcα(2-3)Galβ(1-4)GlcNAcβ(1-3)Galβ(1-4)GlcNAcβ(1-2)[NeuAcα(2-3)Galβ(1-4)GlcNAcβ(1-3)Galβ(1-4)GlcNAcβ(1-6)Manα(1-6)]}-Manβ(1-4)GlcNAcβ(1-4)GlcNAcβ-(Lys-Val-Ala)Asn-Lys-Thr-NH2	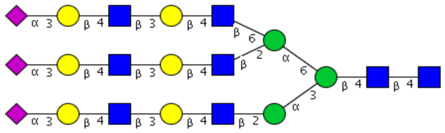
**62**	NeuAcα(2-3)Galβ(1-4)GlcNAcβ(1-3)Galβ(1-4)GlcNAcβ(1-3)Galβ(1-4)GlcNAcβ(1-2)Manα(1-3){NeuAcα(2-3)Galβ(1-4)GlcNAcβ(1-3)Galβ(1-4)GlcNAcβ(1-3)Galβ(1-4)GlcNAcβ(1-2)[NeuAcα(2-3)Galβ(1-4)GlcNAcβ(1-3)Galβ(1-4)GlcNAcβ(1-3)Galβ(1-4)GlcNAcβ(1-6)Manα(1-6)]}-Manβ(1-4)GlcNAcβ(1-4)GlcNAcβ-(Lys-Val-Ala)Asn-Lys-Thr-NH2	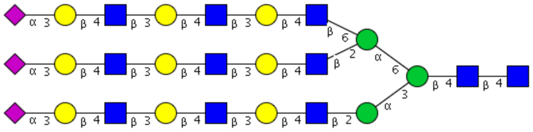
**63**	NeuAcα(2-3)Galβ(1-4)GlcNAcβ(1-3)Galβ(1-4)GlcNAcβ(1-2)Manα(1-3){NeuAcα(2-3)Galβ(1-4)GlcNAcβ(1-3)Galβ(1-4)GlcNAcβ(1-2)[NeuAcα(2-3)Galβ(1-4)GlcNAcβ(1-3)Galβ(1-4)GlcNAcβ(1-6)Manα(1-6)]}-Manβ(1-4)GlcNAcβ(1-4)[Fucα(1-6)]-GlcNAcβ-(Lys-Val-Ala)Asn-Lys-Thr-NH2	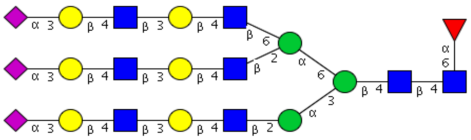

Glycan #	Common Name	Structure
64	NeuAcα(2-3)Galβ(1-4)GlcNAcβ(1-3)Galβ(1-4)GlcNAcβ(1-3)Galβ(1-4)GlcNAcβ(1-2)Manα(1-3){NeuAcα(2-3)Galβ(1-4)GlcNAcβ(1-3)Galβ(1-4)GlcNAcβ(1-3)Galβ(1-4)GlcNAcβ(1-2)[NeuAcα(2-3)Galβ(1-4)GlcNAcβ(1-3)Galβ(1-4)GlcNAcβ(1-3)Galβ(1-4)GlcNAcβ(1-6)Manα(1-6)]}-Manβ(1-4)GlcNAcβ(1-4)[Fucα(1-6)]-GlcNAcβ-(Lys-Val-Ala)Asn-Lys-Thr-NH2	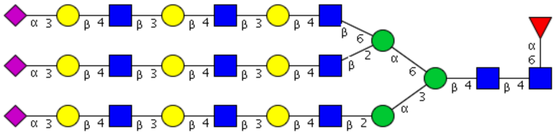
65	Gn/3'SLN/3'SLN-TriN	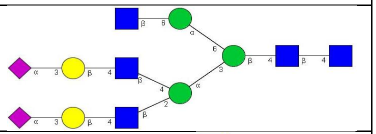
66	NeuAcα(2-3)[GalNAcβ(1-4)]-Galβ(1-4)GlcNAcβ-ethyl-NH2	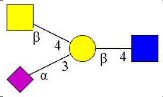
67	NeuAcα(2-3)[GalNAcβ(1-4)]-Galβ(1-4)Glcβ-ethyl-NH2	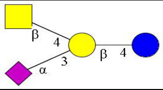
68	Galβ(1-3)GalNAcβ(1-4)[NeuAcα(2-3)]-Galβ(1-4)Glcβ-ethyl-NH2	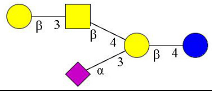
69	NeuAcα(2-3)Galβ(1-4)[Fucα(1-3)]-GlcNAcβ-propyl-NH2	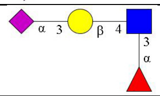
70	NeuAcα(2-3)Galβ(1-3)[Fucα(1-4)]-GlcNAcβ(1-3)Galβ(1-4)[Fucα(1-3)]-GlcNAcβ-ethyl-NH2	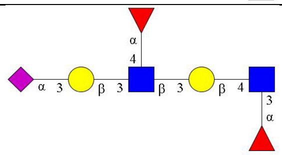
71	NeuAcα(2-3)Galβ(1-4)[Fucα(1-3)]-GlcNAcβ(1-3)Galβ(1-4)[Fucα(1-3)]-GlcNAcβ-ethyl-NH2	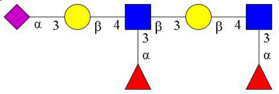
72	NeuAcα(2-3)Galβ(1-4)[Fucα(1-3)]-GlcNAcβ(1-3)Galβ(1-4)[Fucα(1-3)]-GlcNAcβ(1-3)Galβ(1-4)[Fucα(1-3)]-GlcNAcβ-ethyl-NH2	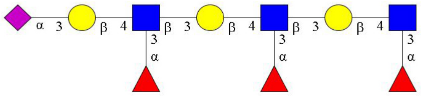
73	NeuAcα(2-3)Galβ(1-4)[Fucα(1-3)]-GlcNAcβ(1-3)Galβ(1-4)[Fucα(1-3)]-GlcNAcβ(1-3)Galβ(1-4)[Fucα(1-3)]-GlcNAcβ(1-3)Galβ(1-3)GalNAcα-Thr-NH2	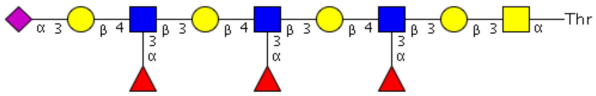
74	NeuAcα(2-3)Galβ(1-4)[Fucα(1-3)]-GlcNAcβ(1-3)Galβ(1-4)[Fucα(1-3)]-GlcNAcβ(1-3)Galβ(1-4)[Fucα(1-3)]-GlcNAcβ(1-3)GalNAcα-Thr-NH2	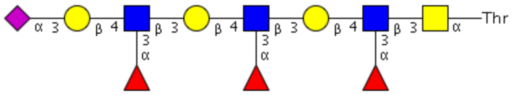
75	NeuAcα(2-3)Galβ(1-4)[Fucα(1-3)]-GlcNAcβ(1-3)Galβ(1-4)[Fucα(1-3)]-GlcNAcβ(1-3)Galβ(1-4)[Fucα(1-3)]-GlcNAcβ(1-3)[NeuAcα(2-3)Galβ(1-4)[Fucα(1-3)]-GlcNAcβ(1-3)Galβ(1-4)[Fucα(1-3)]-GlcNAcβ(1-3)Galβ(1-4)[Fucα(1-3)]-GlcNAcβ(1-6)]-GalNAcα-Thr-NH2	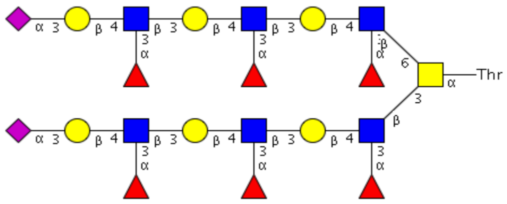

Glycan #	Common Name	Structure
76	NeuAcα(2-3)Galβ(1-4)[Fucα(1-3)]-GlcNAcβ(1-2)Manα(1-3)[NeuAcα(2-3)Galβ(1-4)[Fucα(1-3)]-GlcNAcβ(1-2)Manα(1-6)]-Manβ(1-4)GlcNAcβ(1-4)GlcNAcβ-(Lys-Val-Ala)Asn-(Lys-Thr)NH2	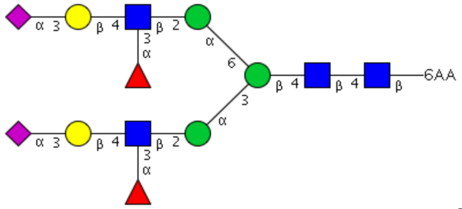
77	NeuAcα(2-6)Galb(1-4)(6S)GlcNacb-ethyl-NH2	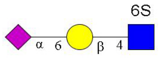
78	NeuAcα(2-6)Galβ(1-4)6-O-sulfo-GlcNAcβ-propyl-NH2	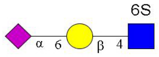
79	NeuAcα(2-6)Galβ(1-4)Glcβ-ethyl-NH2	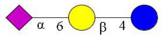
80	NeuAcα(2-6)Galβ(1-4)GlcNAcβ-ethyl-NH2	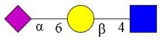
81	NeuAcα(2-6)Galβ(1-4)GlcNAcβ(1-3)Galβ(1-4)GlcNAcβ-ethyl-NH2	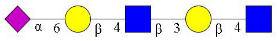
82	NeuAcα(2-6)Galβ(1-4)GlcNAcβ(1-3)Galβ(1-4)GlcNAcβ(1-3)Galβ(1-4)GlcNAcβ-ethyl-NH2	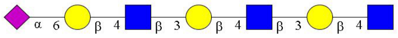
83	NeuAcα(2-6)GalNAcβ(1-4)GlcNAcβ-ethyl-NH2	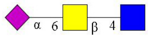
84	NeuAcα(2-6)Galβ(1-4)GlcNAcβ(1-3)Galβ(1-3)GalNAcα-Thr-NH2	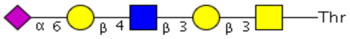
85	NeuAcα(2-6)Galβ(1-4)GlcNAcβ(1-3)Galβ(1-4)GlcNAcβ(1-3)Galβ(1-3)GalNAcα-Thr-NH2	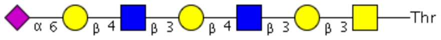
86	NeuAcα(2-6)Galβ(1-4)GlcNAcβ(1-3)Galβ(1-4)GlcNAcβ(1-3)Galβ(1-4)GlcNAcβ(1-3)Galβ(1-3)GalNAcα-Thr-NH2	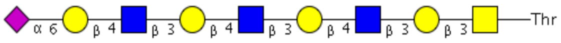
87	NeuAcα(2-6)Galβ(1-4)GlcNAcβ(1-3)Galβ(1-4)GlcNAcβ(1-3)Galβ(1-4)GlcNAcβ(1-3)Galβ(1-4)GlcNAcβ(1-3)Galβ(1-3)GalNAcα-Thr-NH2	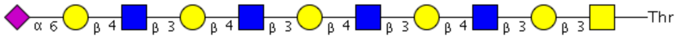
88	NeuAcα(2-6)Galβ(1-4)GlcNAcβ(1-3)Galβ(1-4)GlcNAcβ(1-3)Galβ(1-4)GlcNAcβ(1-3)Galβ(1-4)GlcNAcβ(1-3)Galβ(1-4)GlcNAcβ(1-3)Galβ(1-3)GalNAcα-Thr-NH2	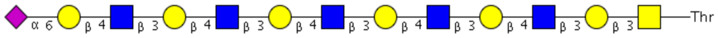
89	NeuAcα(2-6)Galβ(1-4)GlcNAcβ(1-6)[Galβ(1-3)]-GalNAcα-Thr-NH2	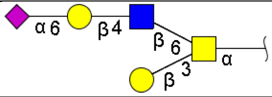
90	NeuAcα(2-6)Galβ(1-4)GlcNAcβ(1-3)Galβ(1-4)GlcNAcβ(1-6)[Galβ(1-3)]-GalNAcα-Thr-NH2	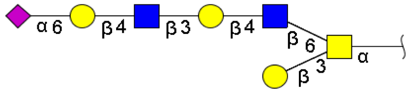
91	NeuAcα(2-6)Galβ(1-4)GlcNAcβ(1-3)Galβ(1-4)GlcNAcβ(1-3)Galβ(1-4)GlcNAcβ(1-6)[Galβ(1-3)]-GalNAcα-Thr-NH2	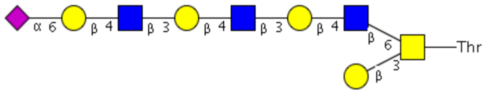
92	NeuAcα(2-6)Galβ(1-4)GlcNAcβ(1-3)Galβ(1-4)GlcNAcβ(1-3)Galβ(1-4)GlcNAcβ(1-3)Galβ(1-4)GlcNAcβ(1-6)[Galβ(1-3)]-GalNAcα-Thr-NH2	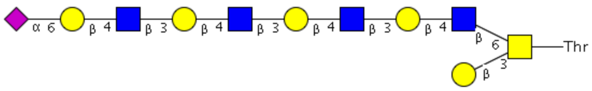

Glycan #	Common Name	Structure
**93**	NeuAcα(2-6)Galβ(1-4)GlcNAcβ(1-3)Galβ(1-4)GlcNAcβ(1-3)Galβ(1-4)GlcNAcβ(1-3)Galβ(1-4)GlcNAcβ(1-3)Galβ(1-4)GlcNAcβ(1-6)[Galβ(1-3)]-GalNAcα-Thr-NH2	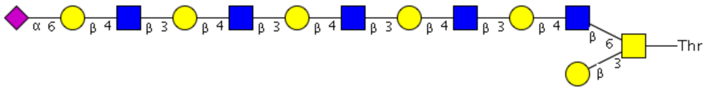
**94**	NeuAcα(2-6)Galβ(1-4)GlcNAcβ(1-3)Galβ(1-4)GlcNAcβ(1-3)Galβ(1-4)GlcNAcβ(1-3)Galβ(1-4)GlcNAcβ(1-6)[NeuAcα(2-6)Galβ(1-4)GlcNAcβ(1-3)Galβ(1-4)GlcNAcβ(1-3)Galβ(1-4)GlcNAcβ(1-3)Galβ(1-3)]-GalNAcα-Thr-NH2	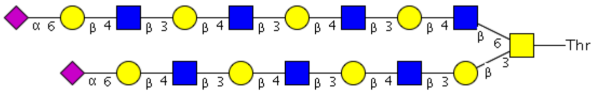
**95**	NeuAcα(2-6)Galβ(1-4)GlcNAcβ(1-3)Galβ(1-4)GlcNAcβ(1-3)Galβ(1-4)GlcNAcβ(1-3)Galβ(1-4)GlcNAcβ(1-3)Galβ(1-4)GlcNAcβ(1-6)[NeuAcα(2-6)Galβ(1-4)GlcNAcβ(1-3)Galβ(1-4)GlcNAcβ(1-3)Galβ(1-4)GlcNAcβ(1-3)Galβ(1-4)GlcNAcβ(1-3)Galβ(1-4)GlcNAcβ(1-3)Galβ(1-3)]-GalNAcα-Thr-NH2	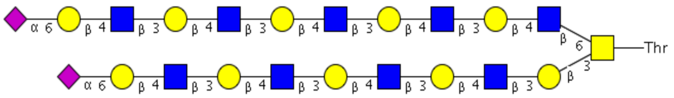
**96**	NeuAcα(2-6)Galβ(1-4)GlcNAcβ(1-3)GalNAcα-Thr-NH2	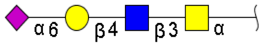
**97**	NeuAcα(2-6)Galβ(1-4)GlcNAcβ(1-3)Galβ(1-4)GlcNAcβ(1-3)GalNAcα-Thr-NH2	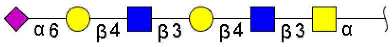
**98**	NeuAcα(2-6)Galβ(1-4)GlcNAcβ(1-3)Galβ(1-4)GlcNAcβ(1-3)Galβ(1-4)GlcNAcβ(1-3)GalNAcα-Thr-NH2	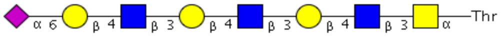
**99**	NeuAcα(2-6)Galβ(1-4)GlcNAcβ(1-3)Galβ(1-4)GlcNAcβ(1-3)Galβ(1-4)GlcNAcβ(1-3)Galβ(1-4)GlcNAcβ(1-3)GalNAcα-Thr-NH2	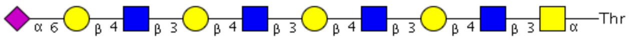
**100**	NeuAcα(2-6)Galβ(1-4)GlcNAcβ(1-3)Galβ(1-4)GlcNAcβ(1-3)Galβ(1-4)GlcNAcβ(1-3)Galβ(1-4)GlcNAcβ(1-3)Galβ(1-4)GlcNAcβ(1-3)Galβ(1-4)GlcNAcβ(1-3)GalNAcα-Thr-NH2	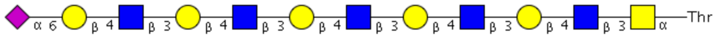
**101**	NeuAcα(2-6)Galβ(1-4)GlcNAcβ(1-3)[NeuAcα(2-6)Galβ(1-4)GlcNAcβ(1-6)]-GalNAcα-Thr-NH2	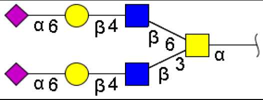
**102**	NeuAcα(2-6)Galβ(1-4)GlcNAcβ(1-3)Galβ(1-4)GlcNAcβ(1-3)[NeuAcα(2-6)Galβ(1-4)GlcNAcβ(1-3)Galβ(1-4)GlcNAcβ(1-6)]-GalNAcα-Thr-NH2	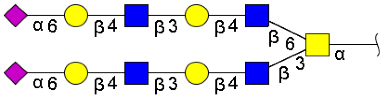
**103**	NeuAcα(2-6)Galβ(1-4)GlcNAcβ(1-3)Galβ(1-4)GlcNAcβ(1-3)Galβ(1-4)GlcNAcβ(1-3)[NeuAcα(2-6)Galβ(1-4)GlcNAcβ(1-3)Galβ(1-4)GlcNAcβ(1-3)Galβ(1-4)GlcNAcβ(1-6)]-GalNAcα-Thr-NH2	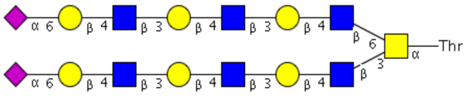
**104**	NeuAcα(2-6)Galβ(1-4)GlcNAcβ(1-3)Galβ(1-4)GlcNAcβ(1-3)Galβ(1-4)GlcNAcβ(1-3)Galβ(1-4)GlcNAcβ(1-3)[NeuAcα(2-6)Galβ(1-4)GlcNAcβ(1-3)Galβ(1-4)GlcNAcβ(1-3)Galβ(1-4)GlcNAcβ(1-3)Galβ(1-4)GlcNAcβ(1-6)]-GalNAcα-Thr-NH2	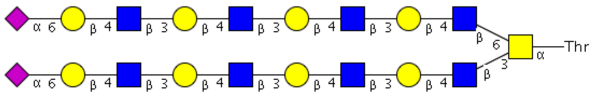

Glycan #	Common Name	Structure
**105**	NeuAcα(2-6)Galβ(1-4)GlcNAcβ(1-3)Galβ(1-4)GlcNAcβ(1-3)Galβ(1-4)GlcNAcβ(1-3)Galβ(1-4)GlcNAcβ(1-3)Galβ(1-4)GlcNAcβ(1-3)[NeuAcα(2-6)Galβ(1-4)GlcNAcβ(1-3)Galβ(1-4)GlcNAcβ(1-3)Galβ(1-4)GlcNAcβ(1-3)Galβ(1-4)GlcNAcβ(1-3)Galβ(1-4)GlcNAcβ(1-6)]-GalNAcα-Thr-NH2	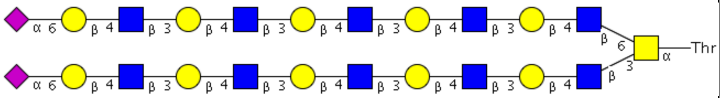
**106**	NeuAcα(2-6)Galβ(1-4)GlcNAcβ(1-3)Galβ(1-4)GlcNAcβ(1-3)Galβ(1-4)GlcNAcβ(1-3)Galβ(1-4)GlcNAcβ(1-6)GalNAcα-Thr-NH2	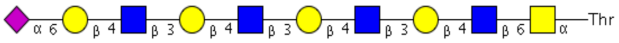
**107**	NeuAcα(2-6)Galβ(1-4)GlcNAcβ(1-3)Galβ(1-4)GlcNAcβ(1-3)Galβ(1-4)GlcNAcβ(1-3)Galβ(1-4)GlcNAcβ(1-3)Galβ(1-4)GlcNAcβ(1-6)GalNAcα-Thr-NH2	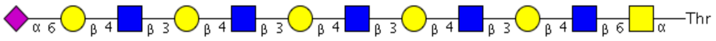
**108**	NeuAcα(2-6)Galβ(1-4)GlcNAcβ(1-3)Galβ(1-4)GlcNAcβ(1-3)Galβ(1-4)GlcNAcβ(1-6)[NeuAcα(2-6)Galβ(1-4)GlcNAcβ(1-3)Galβ(1-4)GlcNAcβ(1-3)] Galβ(1-4)GlcNAcβ-ethyl-NH2	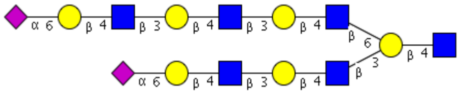
**109**	NeuAcα(2-6)Galβ(1-3)GlcNAcβ(1-3)Galβ(1-4)GlcNAcβ(1-6)[NeuAcα(2-6)Galβ(1-3)GlcNAcβ(1-3)] Galβ(1-4)GlcNAcβ-ethyl-NH2	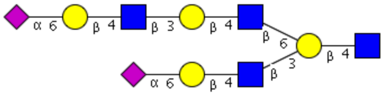
**110**	Galβ(1-4)GlcNAcβ(1-2)Manα(1-3)[NeuAcα(2-6)Galβ(1-4)GlcNAcβ(1-2)Manα(1-6)]-Manβ(1-4)GlcNAcβ(1-4)GlcNAcβ-Asn-NH2	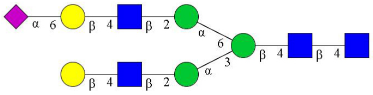
**111**	NeuAcα(2-6)Galβ(1-4)GlcNAcβ(1-2)Manα(1-3)[Galβ(1-4)GlcNAcβ(1-2)Manα(1-6)]-Manβ(1-4)GlcNAcβ(1-4)GlcNAcβ-Asn-NH2	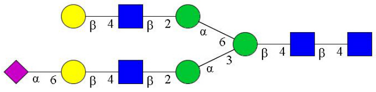
**112**	GlcNAcβ(1-2)Manα(1-3)[NeuAcα(2-6)Galβ(1-4)GlcNAcβ(1-2)Manα(1-6)]-Manβ(1-4)GlcNAcβ(1-4)GlcNAcβ-Asn-NH2	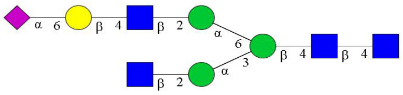
**113**	NeuAcα(2-6)Galβ(1-4)GlcNAcβ(1-2)Manα(1-3)[NeuAcα(2-6)Galβ(1-4)GlcNAcβ(1-2)Manα(1-6)]-Manβ(1-4)GlcNAcβ(1-4)GlcNAcβ-Asn-NH2	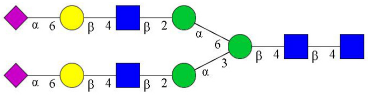
**114**	NeuAcα(2-6)Galβ(1-4)GlcNAcβ(1-3)Galβ(1-4)GlcNAcβ(1-2)Manα(1-3)[NeuAcα(2-6)Galβ(1-4)GlcNAcβ(1-3)Galβ(1-4)GlcNAcβ(1-2)Manα(1-6)]-Manβ(1-4)GlcNAcβ(1-4)GlcNAcβ-Asn-NH2	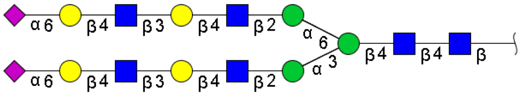
**115**	NeuAcα(2-6)Galβ(1-4)GlcNAcβ(1-3)Galβ(1-4)GlcNAcβ(1-2)Manα(1-3)[NeuAcα(2-6)Galβ(1-4)GlcNAcβ(1-3)Galβ(1-4)GlcNAcβ(1-2)Manα(1-6)]-Manβ(1-4)GlcNAcβ(1-4)GlcNAcβ-(Lys-Val-Ala)Asn-Lys-Thr-NH2	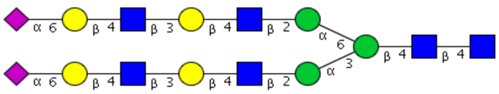

Glycan #	Common Name	Structure
**116**	NeuAcα(2-6)Galβ(1-4)GlcNAcβ(1-3)Galβ(1-4)GlcNAcβ(1-3)Galβ(1-4)GlcNAcβ(1-2)Manα(1-3)[NeuAcα(2-6)Galβ(1-4)GlcNAcβ(1-3)Galβ(1-4)GlcNAcβ(1-3)Galβ(1-4)GlcNAcβ(1-2)Manα(1-6)]-Manβ(1-4)GlcNAcβ(1-4)GlcNAcβ-Asn-NH2	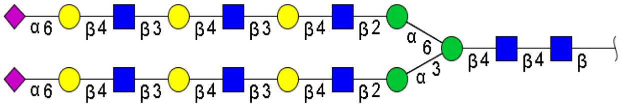
**117**	NeuAcα(2-6)Galβ(1-4)GlcNAcβ(1-3)Galβ(1-4)GlcNAcβ(1-3)Galβ(1-4)GlcNAcβ(1-2)Manα(1-3)[NeuAcα(2-6)Galβ(1-4)GlcNAcβ(1-3)Galβ(1-4)GlcNAcβ(1-3)Galβ(1-4)GlcNAcβ(1-2)Manα(1-6)]-Manβ(1-4)GlcNAcβ(1-4)GlcNAcβ-(Lys-Val-Ala)Asn-Lys-Thr-NH2	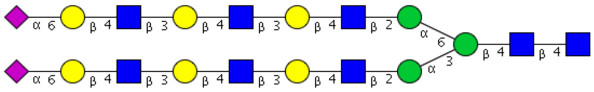
**118**	NeuAcα(2-6)Galβ(1-4)GlcNAcβ(1-3)Galβ(1-4)GlcNAcβ(1-3)Galβ(1-4)GlcNAcβ(1-3)Galβ(1-4)GlcNAcβ(1-2)Manα(1-3)[NeuAcα(2-6)Galβ(1-4)GlcNAcβ(1-3)Galβ(1-4)GlcNAcβ(1-3)Galβ(1-4)GlcNAcβ(1-3)Galβ(1-4)GlcNAcβ(1-2)Manα(1-6)]-Manβ(1-4)GlcNAcβ(1-4)GlcNAcβ-(Lys-Val-Ala)Asn-Lys-Thr-NH2	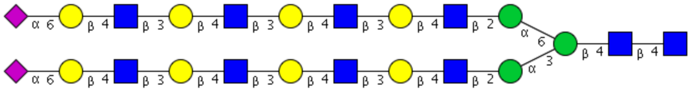
**119**	NeuAcα(2-6)Galβ(1-4)GlcNAcβ(1-3)Galβ(1-4)GlcNAcβ(1-3)Galβ(1-4)GlcNAcβ(1-3)Galβ(1-4)GlcNAcβ(1-3)Galβ(1-4)GlcNAcβ(1-2)Manα(1-3)[NeuAcα(2-6)Galβ(1-4)GlcNAcβ(1-3)Galβ(1-4)GlcNAcβ(1-3)Galβ(1-4)GlcNAcβ(1-3)Galβ(1-4)GlcNAcβ(1-3)Galβ(1-4)GlcNAcβ(1-2)Manα(1-6)]-Manβ(1-4)GlcNAcβ(1-4)GlcNAcβ-(Lys-Val-Ala)Asn-Lys-Thr-NH2	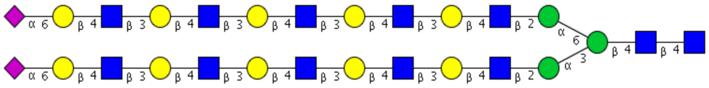
**120**	NeuAcα(2-6)Galβ(1-4)GlcNAcβ(1-3)Galβ(1-4)GlcNAcβ(1-2)Manα(1-3)[NeuAcα(2-6)Galβ(1-4)GlcNAcβ(1-3)Galβ(1-4)GlcNAcβ(1-2)Manα(1-6)]-Manβ(1-4)GlcNAcβ(1-4)[Fucα(1-6)]-GlcNAcβ-(Lys-Val-Ala)Asn-Lys-Thr-NH2	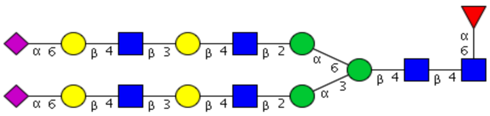
**121**	NeuAcα(2-6)Galβ(1-4)GlcNAcβ(1-3)Galβ(1-4)GlcNAcβ(1-3)Galβ(1-4)GlcNAcβ(1-2)Manα(1-3)[NeuAcα(2-6)Galβ(1-4)GlcNAcβ(1-3)Galβ(1-4)GlcNAcβ(1-3)Galβ(1-4)GlcNAcβ(1-2)Manα(1-6)]-Manβ(1-4)GlcNAcβ(1-4)[Fucα(1-6)]-GlcNAcβ-(Lys-Val-Ala)Asn-Lys-Thr-NH2	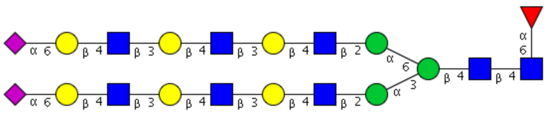

Glycan #	Common Name	Structure
**122**	NeuAcα(2-6)Galβ(1-4)GlcNAcβ(1-3)Galβ(1-4)GlcNAcβ(1-3)Galβ(1-4)GlcNAcβ(1-3)Galβ(1-4)GlcNAcβ(1-2)Manα(1-3)[NeuAcα(2-6)Galβ(1-4)GlcNAcβ(1-3)Galβ(1-4)GlcNAcβ(1-3)Galβ(1-4)GlcNAcβ(1-3)Galβ(1-4)GlcNAcβ(1-2)Manα(1-6)]-Manβ(1-4)GlcNAcβ(1-4)[Fucα(1-6)]-GlcNAcβ-(Lys-Val-Ala)Asn-Lys-Thr-NH2	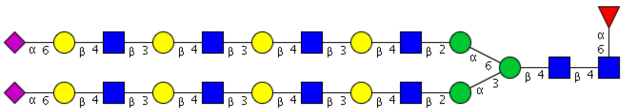
**123**	NeuAcα(2-6)Galβ(1-4)GlcNAcβ(1-3)Galβ(1-4)GlcNAcβ(1-2)Manα(1-3){NeuAcα(2-6)Galβ(1-4)GlcNAcβ(1-3)Galβ(1-4)GlcNAcβ(1-2)[NeuAcα(2-6)Galβ(1-4)GlcNAcβ(1-3)Galβ(1-4)GlcNAcβ(1-6)Manα(1-6)]}-Manβ(1-4)GlcNAcβ(1-4)GlcNAcβ-(Lys-Val-Ala)Asn-Lys-Thr-NH2	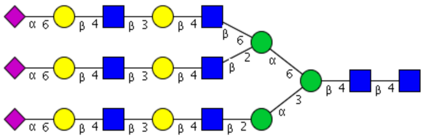
**124**	NeuAcα(2-6)Galβ(1-4)GlcNAcβ(1-3)Galβ(1-4)GlcNAcβ(1-2)Manα(1-3){NeuAcα(2-6)Galβ(1-4)GlcNAcβ(1-3)Galβ(1-4)GlcNAcβ(1-2)[NeuAcα(2-6)Galβ(1-4)GlcNAcβ(1-3)Galβ(1-4)GlcNAcβ(1-6)Manα(1-6)]}-Manβ(1-4)GlcNAcβ(1-4)[Fucα(1-6)]-GlcNAcβ-(Lys-Val-Ala)Asn-Lys-Thr-NH2	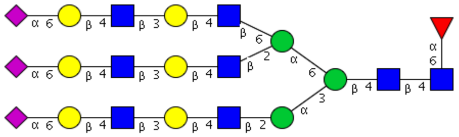
**125**	NeuAcα(2-6)Galβ(1-4)GlcNAcβ(1-3)Galβ(1-4)GlcNAcβ(1-3)Galβ(1-4)GlcNAcβ(1-2)Manα(1-3){NeuAcα(2-6)Galβ(1-4)GlcNAcβ(1-3)Galβ(1-4)GlcNAcβ(1-3)Galβ(1-4)GlcNAcβ(1-2)[NeuAcα(2-6)Galβ(1-4)GlcNAcβ(1-3)Galβ(1-4)GlcNAcβ(1-3)Galβ(1-4)GlcNAcβ(1-6)Manα(1-6)]}-Manβ(1-4)GlcNAcβ(1-4)[Fucα(1-6)]-GlcNAcβ-(Lys-Val-Ala)Asn-Lys-Thr-NH2	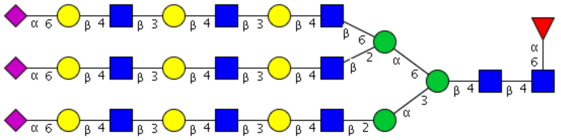
**126**	LN/6'SLN/6'SLN-TriN	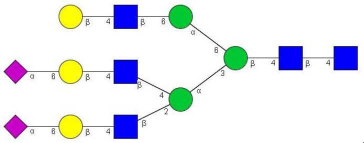
**127**	6'SLN/LeX/LeX-TriN	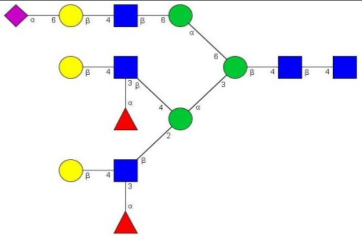
**128**	6'SLNLN/LeX/LeX-TriN	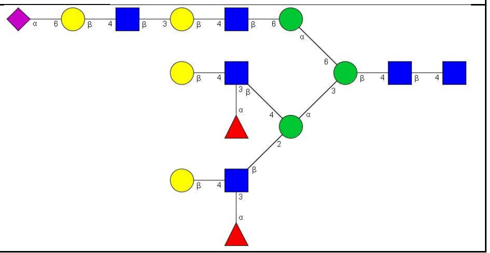
